# Dynamic Changes in Rhizosphere Microecology of Ephemeroid *Ferula* and Their Effects on Soil Quality in Arid Regions

**DOI:** 10.3390/plants15182846

**Published:** 2026-09-17

**Authors:** Hanli Dang, Zhilin Zhang, Aifang Ma, Tao Zhang

**Affiliations:** Ministry Key Laboratory of Xinjiang Phytomedicine Resource and Utilization, Key Laboratory of Oasis Town and Mountain-Basin System Ecology of Bingtuan, College of Life Sciences, Shihezi University, Shihezi 832003, China; 13199931067@163.com (H.D.); 19119904935@163.com (Z.Z.); 13993467164@163.com (A.M.)

**Keywords:** ephemeroid plants, soil physicochemical properties, soil microbial community, soil quality

## Abstract

As early-spring pioneer ephemeroids in arid and semi-arid regions, *Ferula* species exert irreplaceable ecological functions through distinctive life-history strategies. However, their impacts on soil microhabitats and soil quality remain unclear. Taking *Ferula* species (*Ferula feruloides*, *Ferula lehmannii*, *Ferula krylovii*, and *Ferula sinkiangensis*) as the research subject, this study conducted a comprehensive analysis of the soil abiotic (physicochemical properties) and biological (microbial (bacterial/fungal) community diversity, composition, structure, and co-occurrence networks) factors as well as their correlations in the *Ferula*-planted areas (EX) and the unplanted areas (CK), and comprehensively assessed the soil quality index (SQI). Results showed the following: (1) The EX significantly affected soil nutrient contents and microbial community diversity indices (*p* < 0.05), with interspecific specificity and temporal dynamics. (2) The EX enhanced the stability/connectivity of microbial co-occurrence networks but reduced connection density (opposite to CK). (3) Changes in soil physicochemical factors were correlated with variations in the composition and distribution of rhizosphere microbial communities in *Ferula* species, with bacteria showing closer associations with soil nutrients and fungi with soil properties. Additionally, rhizosphere bacterial diversity indices explained the greatest proportion of SQI improvement. This study clarifies relationships of *Ferula* to the “soil−microbe−quality” continuum, providing scientific support for arid-region soil remediation, vegetation protection, and ecosystem stability.

## 1. Introduction

Ecosystems in arid and semi-arid regions are inherently vulnerable [1]. Aggravated by poor soil fertility, nutrient depletion, and water scarcity, soil degradation has become a critical bottleneck for regional ecological security and agricultural sustainability [2,3]. Vegetation restoration is key to improving soil quality and ecosystem stability, as plants optimize soil nutrient structure, moisture conditions, and biological activity via life processes [4,5]. Ephemeroid plants—arid early-spring pioneers with a “spring rapid growth, summer drought dormancy” strategy—exhibit short growth cycles and high resource use efficiency [6,7]. Through root expansion, litter decomposition, and exudate release, they activate rhizosphere microenvironments, regulate soil physicochemical properties and microbial communities, and serve as key contributors to nutrient cycling in arid soils [8]. Studying their rhizosphere shaping effects deepens understanding of plant−soil−microbe interactions and supports arid-region ecological management.

Soil microbial communities drive material cycling and nutrient turnover, underpinning biodiversity and terrestrial ecosystem functions [9,10]. As the core interface of soil-plant system, rhizosphere microbial dynamics are tightly linked to soil quality and plant adaptability [11], with their composition closely coupled to environmental factors [12]. For example, soil properties shape bacterial communities [13], while pH, SOC, and EC drive fungal differentiation [14]. Furthermore, the soil C:N:P ratio regulates soil biogeochemical processes, including mineralization/immobilization, nitrification/denitrification, and plant uptake/release, which affect the direction and efficiency of the transformation of soil nitrogen and phosphorus from organic to inorganic forms [15]. This association reflects plants modifying rhizospheres to select adaptive microbes, forming a stable plant−microbe−environment system [13,16]. Deciphering ephemeroid plants’ effects on rhizosphere soil microhabitats and their drivers clarifies plant–microbe adaptations, supporting arid-region vegetation enhancement and ecosystem restoration.

Soil quality refers to the comprehensive embodiment of soil physical, chemical, and biological properties, and this indicator can reflect changes in ecosystem stability [17,18]. As an important biological factor driving soil quality, vegetation can regulate the processes of organic matter cycling and energy flow in ecosystems through the interaction between underground plant roots and microorganisms, which is a lengthy and complex process. The temporal dynamics of plant-soil microbial communities are typically studied via natural land restoration chronosequences and different developmental stages of plant root microbiomes [19,20]. Multiple studies have shown that root-associated microbial communities are highly dynamic, strongly influenced by plant variety, growth status, and developmental stage [21,22]. Their effects are mainly mediated by root exudates regulating microbial growth, indicating that plants can actively modify their microbiomes throughout their life cycles. Changes in the structure and function of rhizosphere soil microbiomes and their associations with habitats have been used to decipher the adaptive mechanisms of plants in arid and semi-arid regions (e.g., *Alhagi camelorum* Fisch., *Tamarix ramosissima* Ledeb., *Calligonum mongolicum* Turcz., *Stipa breviflora* Griseb, and *Triticum aestivum*) [11,23]. However, research on the temporal dynamics of rhizosphere microbial communities in ephemeroid plants remains insufficient.

*Ferula* plants are a unique ephemeroid group endemic to arid and semi-arid regions and characteristic medicinal plants in Northwest China, with both ecological and economic value. Their wild populations have demonstrated adaptability to barren soils and potential for microhabitat improvement during long-term adaptation to arid habitats [24]. As medicinal plants, *Ferula* can produce various natural active substances and volatile organic compounds (e.g., sesquiterpenoids, coumarins, sulfur-containing compounds, polysaccharides, and aromatic compounds) in their roots and rhizomes. Processes such as root exudate release and residue decomposition can alter soil nutrient availability and microbial community structure through rhizosphere effects, thereby influencing the soil microenvironment [25,26]. However, current research on *Ferula* mainly focuses on resource protection, medicinal component extraction, pharmacological activity, and single-habitat surveys.

With the dual medicinal and ecological values of *Ferula* species gaining increasing recognition, the scale of their artificial cultivation has expanded progressively. However, the coupling relationship among “cultivation practices, soil microhabitats, and soil quality” remains poorly understood. This knowledge gap not only constrains the in-depth elucidation of the ecological functions of ephemeroid plants but also impedes the precise deployment of *Ferula*-based soil remediation technologies in arid regions. Therefore, systematic investigations into the reshaping patterns of rhizosphere soil microhabitats induced by *Ferula* cultivation, as well as the clarification of its regulatory effects on soil quality in arid areas, will not only fill the research void in rhizosphere ecological processes of ephemeroid plants but also provide robust theoretical support and scientific underpinnings for the synergistic development of sustainable utilization of medicinal plant resources and soil ecological remediation in arid zones.

Currently, there is a lack of systematic studies investigating how *Ferula* species influence the rhizosphere microenvironment and soil quality. Thus, this study systematically explores the remodeling process of rhizosphere soil microenvironments by *Ferula* and clarifies their regulatory effects on soil quality in arid regions. It takes four *Ferula* species (typical ephemeroid plants in arid and semi-arid regions) as research objects, and from the perspectives of soil physicochemical properties, changes in bacterial and fungal community diversity/composition/structure/function, and community−environment associations, systematically reveals the regulatory effects of these ephemeroid plants on arid soil microecology throughout their growth cycle (seedling, mature, senescent stages). This study aims to deepen the understanding of the role of medicinal ephemeroid plants in soil remediation, vegetation reconstruction, and sustainable ecosystem development in arid and semi-arid regions, providing theoretical support for ecological governance in relevant areas.

## 2. Results

### 2.1. Cultivation of Ferula Plants Alters Soil Biotic and Abiotic Factors

**Figure 1:**
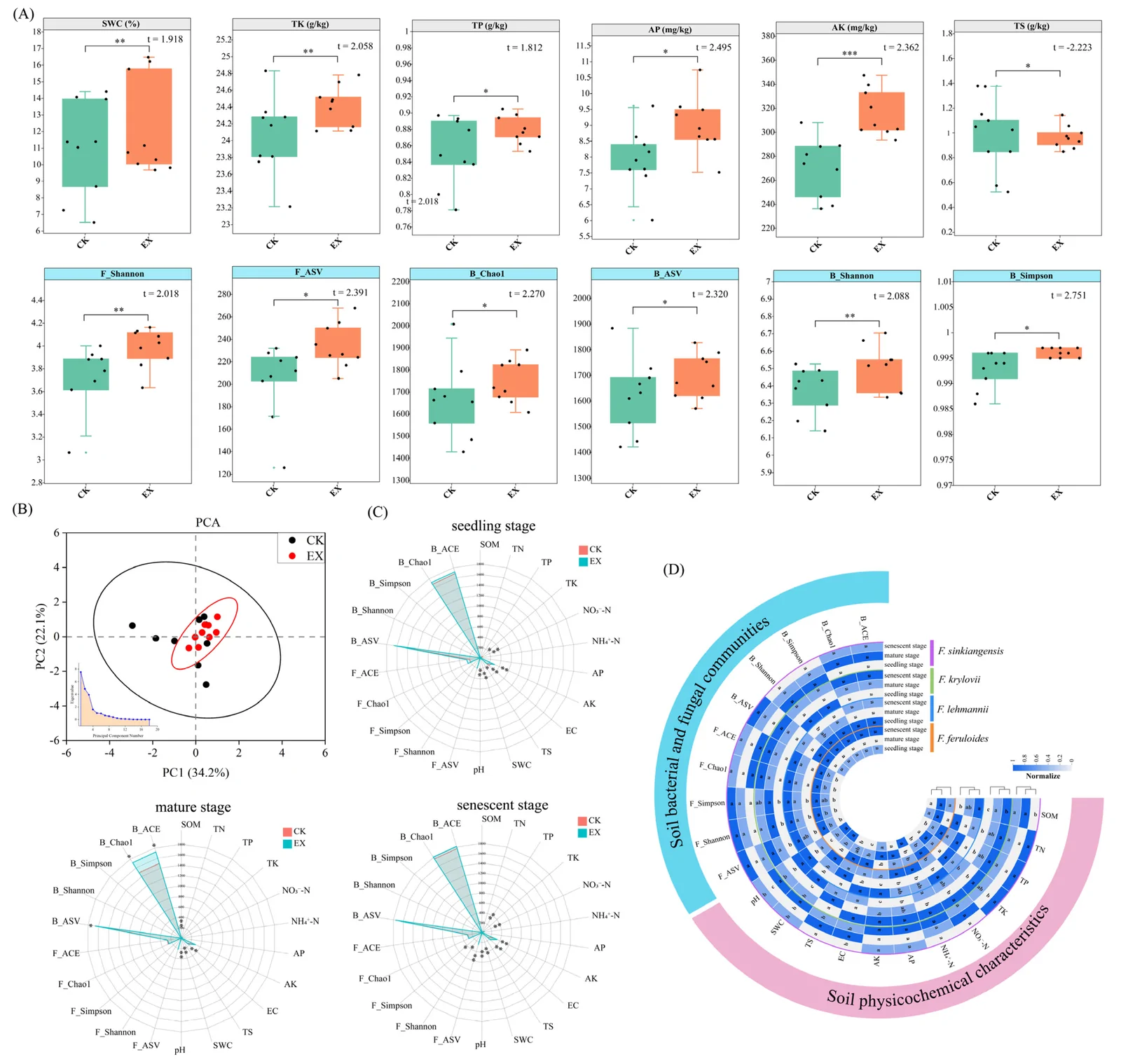
Analysis of soil physicochemical properties and microbial diversity indices. (**A**) Comparative analysis of soil physicochemical properties and microbial diversity indices between *Ferula*-planted (EX) and unplanted (CK) areas (n = 9). * means *p* < 0.05; ** means *p* < 0.01; *** means *p* < 0.001, representing statistically significant differences between groups. (**B**) Principal component analysis of soil factors in the EX and CK groups. (**C**) Radar charts of soil factors in the EX and CK groups at different growth stages. (**D**) Comparison of soil factors across Ferula growth stages. * indicates a significant statistical difference between the EX and CK groups (*p* < 0.05). Different lowercase letters indicate significant differences (*p* < 0.05). Abbreviations: SOM—soil organic matter; TN—total nitrogen; TP—total phosphorus; TK—total potassium; AK—available potassium; NO_3_^−^-N—nitrate nitrogen; NH_4_^+^-N—ammonium nitrogen; AP—available phosphorus; EC—electrical conductivity; TS—total salt; SWC—soil water content; F_ASV, F_Shannon, F_Simpson, F_Chao1, and F_ACE indicate soil fungal diversity indices; B_ASV, B_Shannon, B_Simpson, B_Chao1, and B_ACE indicate soil bacterial diversity indices.

ANOVA results revealed that the cultivation of *Ferula* plant (*Ferula feruloides* (Steud.) Korovin, *Ferula lehmannii* Boiss, *Ferula krylovii* Korovin, and *Ferula sinkiangensis* K. M. Shen) significantly altered soil biotic and abiotic factors (*p* < 0.05, Table S1). Specifically, both soil physicochemical properties, including SWC (11.7%), TK (1.5%), TP (3.2%), AP (14%), and AK (17%), and microbial community diversity indices, including F_Shannon (12.6%), F_ASV (15.5%), B_Chao1 (5%), B_ASV (4.6%), B_Shannon (2%), and B_Simpson (0.3%), in *Ferula*-planted (EX) soils were significantly higher than those in the unplanted control (CK) group, while total salinity (TS, 2.3%) was markedly lower (*p* < 0.05; Figure 1A). The principal component analysis (PCA) revealed that the *Ferula*-planted (EX) soil and unplanted (CK) soil had a separation degree of 56.3%, and could be clearly distinguished into the EX group and CK group, respectively (Figure 1B). Soil biotic and abiotic factors are significantly affected by the growth stage of *Ferula* plants (*p* < 0.05, Table S1). As shown in Figure 1C, at the seedling stage, the EX group exhibited significantly higher TK, AK, SWC, and pH, whereas contents of NO_3_^−^-N, EC, and TS were significantly lower than those in the CK group (*p* < 0.05). At the mature stage, EC, B_ASV, B_Chao1, and B_ACE were markedly increased in the EX group, while SOM, SWC, and pH were decreased (*p* < 0.05). At the senescent stage, the EX group had significantly higher TP, NO_3_^−^-N, AK, EC, SWC, F_ASV, F_Shannon, and F_Simpson, along with a lower pH relative to the CK group (*p* < 0.05).

ANOVA results revealed significant differences in soil biotic and abiotic factors among different *Ferula* species (*p* < 0.05, Table S2, Figure S1). More importantly, the rhizosphere biotic and abiotic factors of *Ferula* species differed significantly across growth stages (*p* < 0.05, Figure 1D): ① *F. feruloides*: NH_4_^+^-N, SWC, and pH peaked at the seedling stage; SOM, TN, TK, AP, and AK peaked at the mature stage; TP, NO_3_^−^-N, EC, TS, and microbial diversity indices peaked at the senescent stage. ② *F. lehmannii*: TN, NH_4_^+^-N, AP, SWC, and bacterial diversity indices peaked at the seedling stage; TP, TK, and TS peaked at the mature stage; SOM, NO_3_^−^-N, AK, EC, pH, and fungal diversity indices peaked at the senescent stage. ③ *F. krylovii*: most indices (SOM, TN, TP, AP, AK, and microbial richness/ASV) followed senescent > mature > seedling; SWC, pH, B_Shannon, and B_Simpson followed seedling > senescent > mature. ④ *F. sinkiangensis*: NH_4_^+^-N, AP, AK, SWC, pH, F_Chao1, F_ACE, B_Shannon, and B_Simpson peaked at the seedling stage; SOM, NO_3_^−^-N, EC, TS, B_Chao1, and B_ACE peaked at the mature stage; TN, TP, TK, F_ASV, F_Shannon, F_Simpson, and B_ASV peaked at the senescent stage. Cluster analysis showed that rhizosphere soil properties of *Ferula* (except *F. feruloides*) could be divided into two groups: seedling stage vs. mature + senescent stages. *F. feruloides* showed seedling + mature stages vs. senescent stage.

### 2.2. Cultivation of Ferula Plants Affects Soil Microbial Communities

#### 2.2.1. Distribution and Structure of Soil Microbial Communities

**Figure 2:**
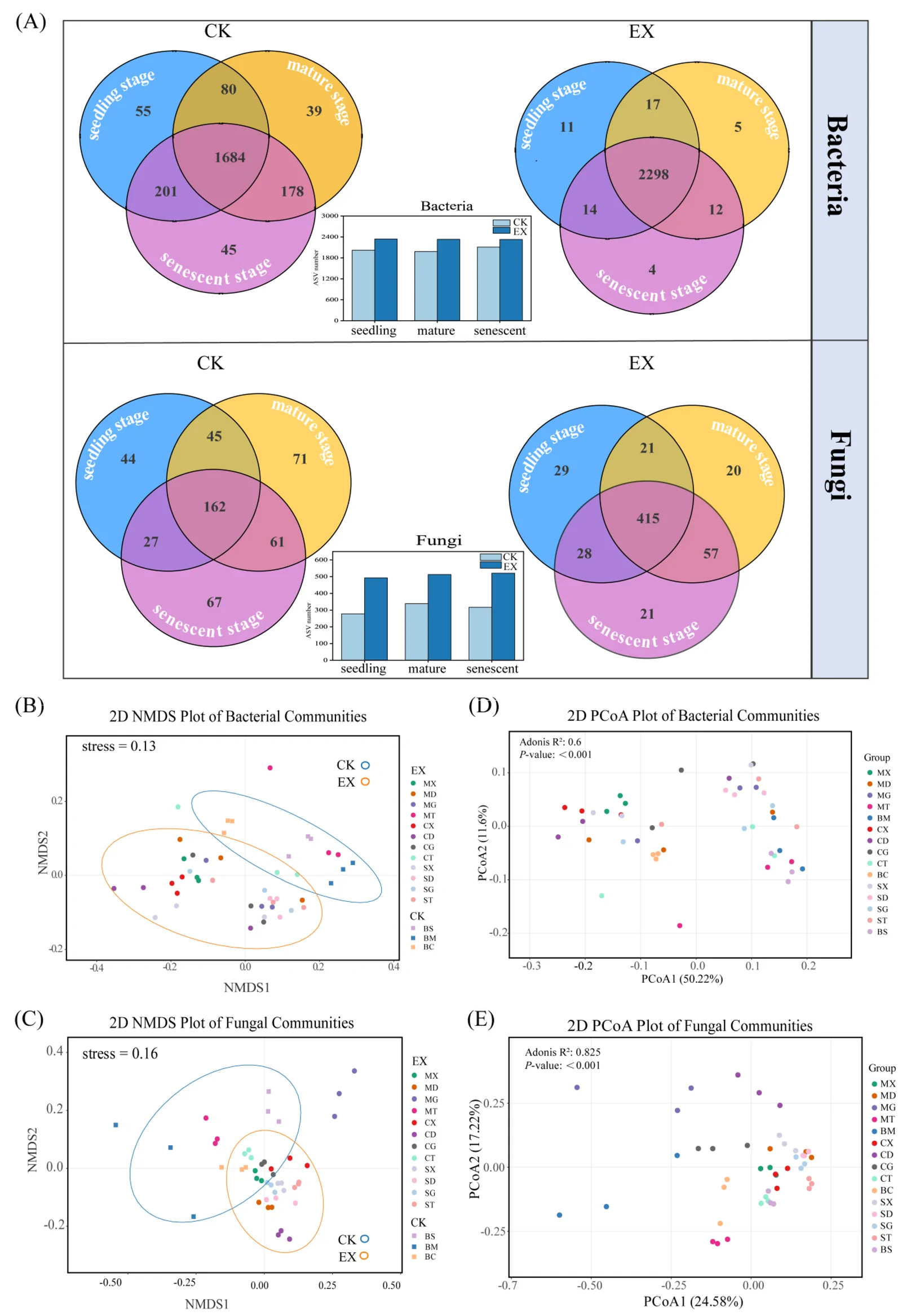
Analysis of the soil microbial community distribution and structure. (**A**) Venn diagram based on ASVs showing shared and unique ASVs of bacterial and fungal communities among groups. (**B**,**C**) NMDS analysis of bacteria and fungi between the CK and EX groups, respectively. (**D**,**E**) PCoA analysis of bacteria and fungi among all groups, respectively. Adonis is a method used to test whether the differences in microbial community composition among different groups are statistically significant. R^2^ represents the proportion of sample variation explained by the grouping factor, and a higher R^2^ value indicates that grouping can explain a larger proportion of the variation. Group codes: first letter (M: seedling stage; C: mature stage; S: senescent stage); second letter (X: *F. sinkiangensis*; D: *F. feruloides*; G: *F. lehmannii*; T: *F. krylovii*). For example, MD = *F. feruloides* at the mature stage; BM, BC, BS = CK samples at the seedling, mature, and senescent stages, respectively.

Venn diagrams showed that shared ASVs of bacterial communities were 1684 (CK) and 2298 (EX), and fungal communities were 162 (CK) and 415 (EX). Across all growth stages, EX had significantly more shared ASVs and fewer unique ASVs than CK (Figure 2A).

NMDS analysis indicated significant differences in bacterial (stress = 0.13) and fungal (stress = 0.16) communities between CK and EX (Figure 2B,C), suggesting *Ferula* cultivation altered microbial community structure. PCoA analysis based on weighted Unifrac distances further showed species and growth stage significantly affected community similarity (Figure 2D,E). For the bacterial community, samples clustered separately along PCoA1 (50.22%) and PCoA2 (11.6%) (R^2^ = 0.6, *p* < 0.001), with samples collectively accounted for 61.82% of variation. For the fungal community, samples clustered separately along PCoA1 (24.58%) and PCoA2 (17.22%) (R^2^ = 0.825, *p* < 0.001), with samples collectively accounting for 41.8% of variation.

#### 2.2.2. Composition of Soil Microbial Communities

**Figure 3:**
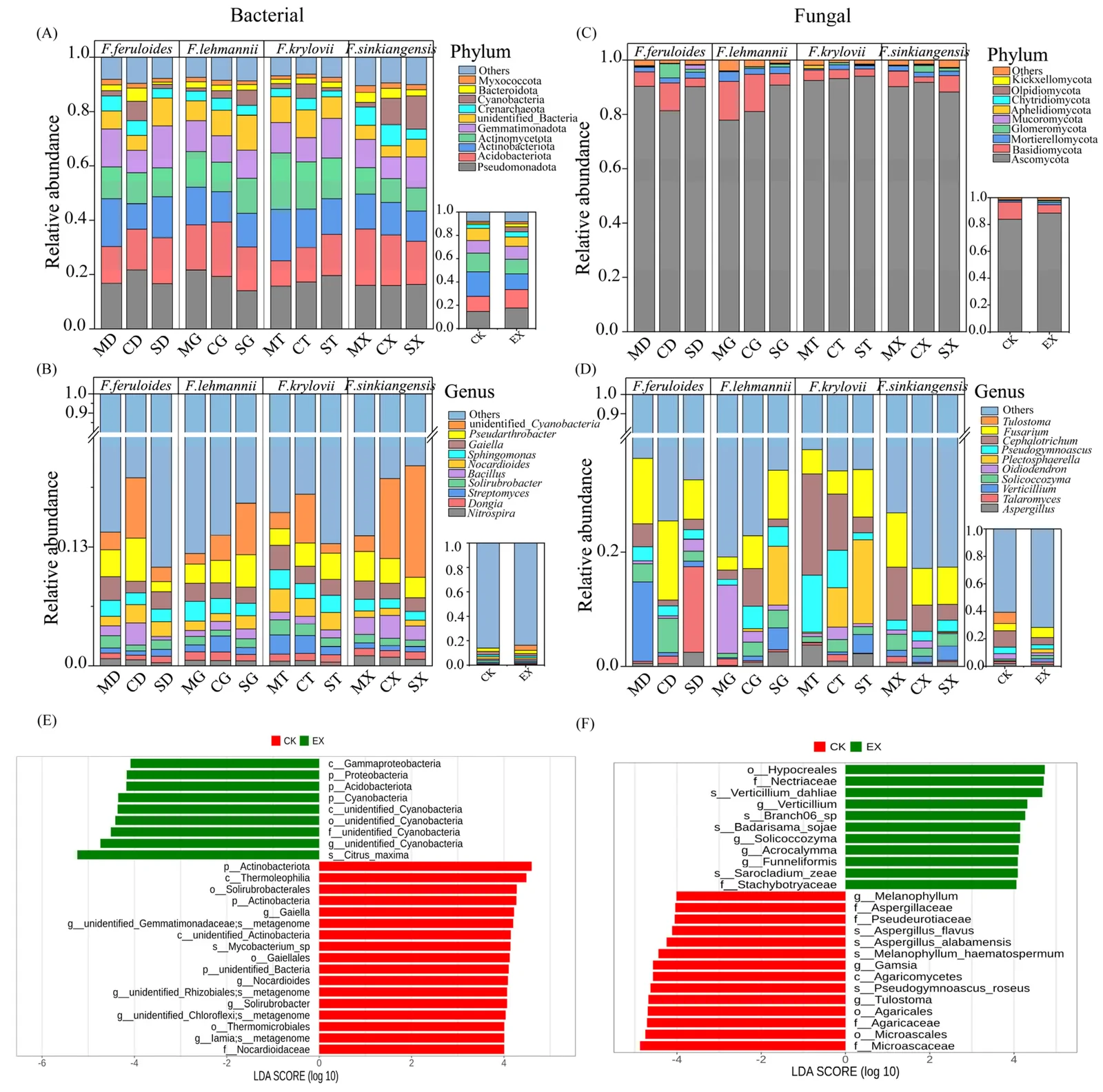
Effects of *Ferula* planting on bacterial and fungal community composition. (**A**,**B**) Relative abundances (top 10) of the bacterial community at phylum and genus levels, respectively. (**C**,**D**) Relative abundances (top 10) of the fungal community at phylum and genus levels, respectively. (**E**,**F**) LDA value distribution bar charts (based on ASVs) showing species with significant abundance differences among groups. Group codes are the same as in Figure 2.

For the bacterial community, 34 phyla, 69 classes, 143 orders, 255 families, 524 genera, and 275 species were annotated. Dominant phyla (Figure 3A) included Pseudomonadota (14.021–21.709%), Acidobacteriota (9.310–20.782%), Actinobacteriota (9.347–18.920%), Actinomycetota (8.511–20.796%), and Gemmatimonadota (8.012–15.467%). At the genus level (Figure 3B), unidentified_Cyanobacteria (1.044–12.165%) was dominant, followed by *Pseudarthrobacter* (1.103–4.715%) and *Gaiella* (1.271–2.684%). ANOVA showed that *Ferula* species, growth stage, or their interaction significantly affected the relative abundance of main dominant bacterial taxa (*p* < 0.05, Table S3); *Ferula* cultivation increased Pseudomonadota, Cyanobacteria, Bacteroidota, and unidentified_Cyanobacteria, while decreasing those of Actinomycetota, *Solirubrobacter*, *Nocardioides*, and *Gaiella* (*p* < 0.05, Table S3).

For the fungal community, 12 phyla, 31 classes, 73 orders, 130 families, 168 genera, and 147 species were annotated. Dominant phyla (Figure 3C) were Ascomycota (77.898–94.056%), Basidiomycota (2.054–14.257%), Mortierellomycota (0.498–3.549%), and Glomeromycota (0.084–5.164%). *Fusarium* (2.275–13.833%) was dominant at the genus level (Figure 3D), with genus dominance varying by sample. ANOVA indicated significant effects of *Ferula* species/growth stage on main fungal taxa (*p* < 0.05, Table S3), and *Ferula* increased Kickxellomycota, Glomeromycota, Mortierellomycota, *Verticillium*, *Solicoccozyma*, and *Plectosphaerella* (*p* < 0.05, Table S3).

LDA analyses showed biomarkers with differences among groups (Figure S2). LEfSe analysis identified 26 bacterial biomarkers (9 in EX, 17 in CK) and 25 fungal biomarkers (11 in EX, 14 in CK) (Figure 3E,F). Specifically, the main dominant bacteria phyla and genera (Pseudomonadota, Acidobacteriota, Cyanobacteria, and unidentified_Cyanobacteria) were the biomarkers with significantly higher abundances in the EX group. At the fungal genus level, EX had four unique biomarkers (*Verticillium, Solicoccozyma*, *Acrocalymma*, and *Funneliformis*) and CK had three unique biomarkers (*Melanophyllum*, *Gamsia*, and *Tulostoma*).

#### 2.2.3. Microbial Community Co-Occurrence Network

**Figure 4:**
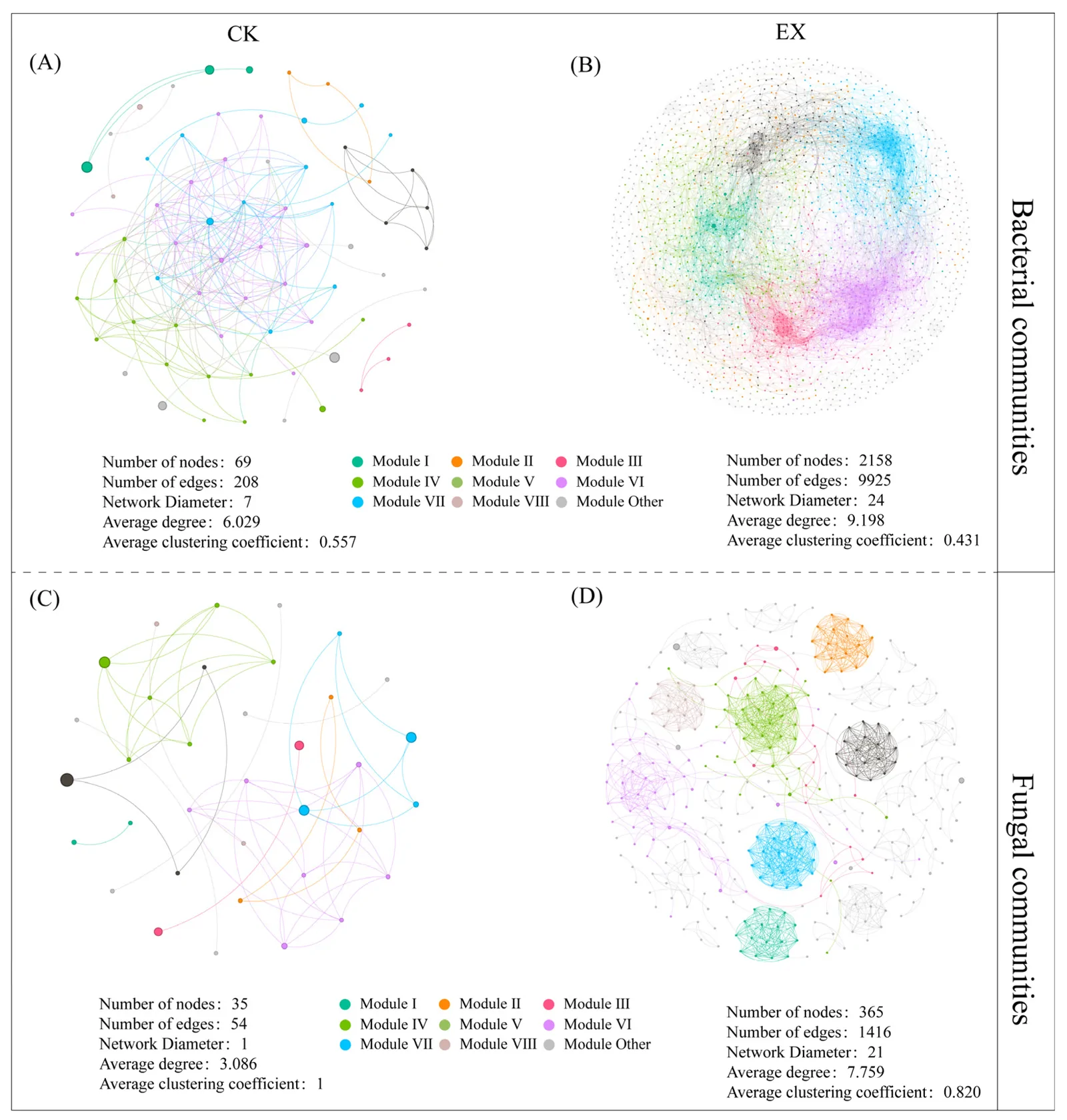
(**A**–**D**) Effects of *Ferula* species on soil bacterial and fungal co-occurrence networks. Modules are distinguished by different colors; each node represents an ASV (a higher degree value means more connections). Edges indicate correlations (r > 0.8, *p* < 0.05), with the thickness positively correlated with the absolute correlation coefficient; red indicates positive correlation, and blue indicates negative correlation.

Co-occurrence networks showed distinct topological differences between EX and CK. For the bacterial network, CK had 6 dominant interacting phyla; EX had 23 dominant interacting phyla (Table S4). EX showed a > 90% increase in the number of nodes/edges and a 34.45% increase in the average degree (Figure 4A,B). For the fungal network, CK had five dominant interacting phyla; EX had nine dominant interacting phyla (Table S4). EX showed a > 90% increase in the number of nodes/edges and a 60.56% increase in average degree (Figure 4C,D). Both networks in EX had an ~ 20% lower average clustering coefficient than CK.

#### 2.2.4. Changes in the Functions of Soil Microbial Communities

**Figure 5:**
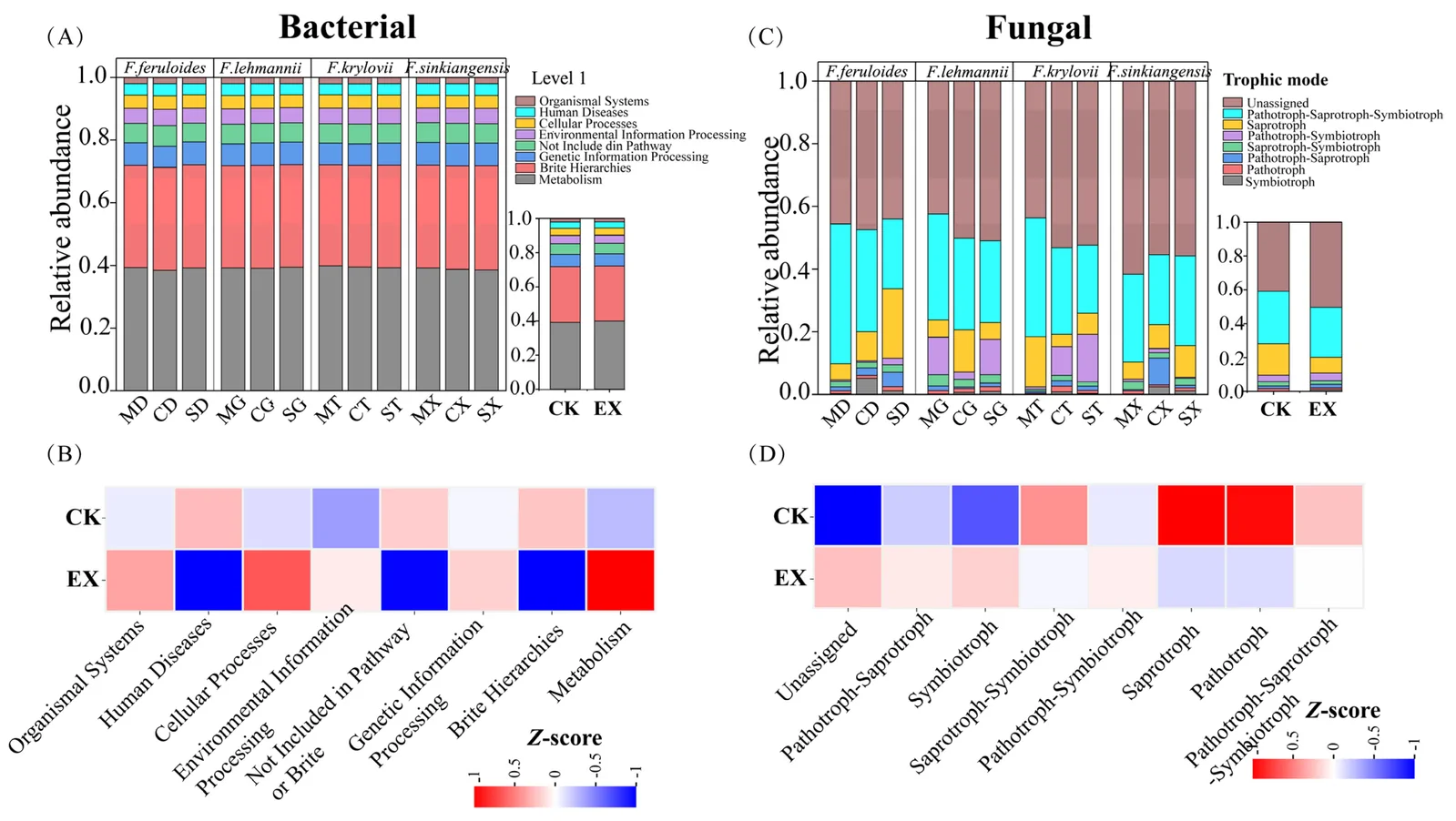
Functional prediction analysis of soil microorganisms among groups. (**A**,**C**) Bar charts showing relative abundances of bacterial (PICRUSt2) and fungal (trophic modes) functional annotations across samples (group codes are the same as those in Figure 2). (**B**,**D**) Clustering heatmaps of bacterial and fungal functional annotations based on Z-scores (red indicates higher relative abundance; blue indicates lower relative abundance).

For the bacterial community, all predicted functional genes at the KEGG L1 classification level mainly included the following categories: Metabolism (39.286%), Brite Hierarchies (32.639%), and Genetic Information Processing (7.084%) (Figure 5A). Z-score normalization showed EX had higher abundances of genes related to Organismal Systems, Cellular Processes, Environmental Information Processing, Genetic Information Processing, and Metabolism than CK (Figure 5B). For the fungal community, all predicted functional genes at the trophic mode level mainly included Pathotroph-Saprotroph-Symbiotroph (29.848%) and Saprotroph (11.087%) (Figure 5C). The heatmap further revealed that the functional groups, namely Pathotroph-Saprotroph, Symbiotroph, and Pathotroph-Symbiotroph, were more clustered in the EX than in the CK (Figure 5D).

### 2.3. Associations Between Environmental Factors and Microbial Communities

**Figure 6:**
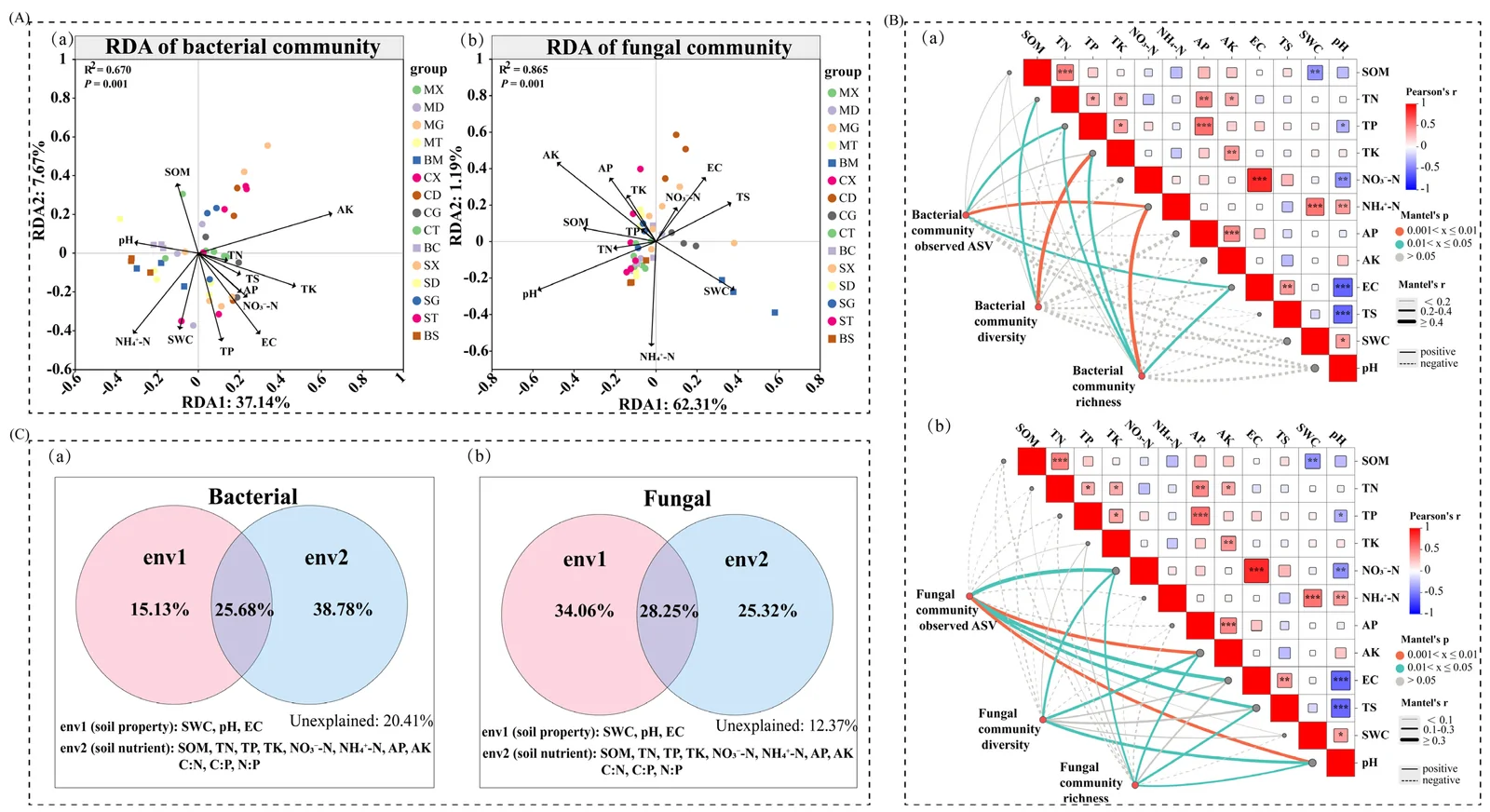
Effects of soil physicochemical factors on the bacterial and fungal communities. (**A**) db-RDA analysis showing relationships between microorganisms and environmental factors. (**a**,**b**) represent bacteria and fungi respectively (group codes are the same as those in Figure 2). (**B**) Pearson correlation analysis of soil factors and Mantel test for their relationships with the bacterial (**a**) and fungal (**b**) community diversity/richness (* *p* < 0.05; ** *p* < 0.01; *** *p* < 0.001). (**C**) VPA analysis of relationships between microbial communities and soil nutrients/properties. Abbreviations are the same as in Figure 1.

db-RDA based on Bray−Curtis dissimilarity showed soil physicochemical factors explained 44.81% (bacteria) and 63.5% (fungi) of community variation, indicating fungi were more affected by physicochemical factors (Figure 6A). For bacteria, the critical factors were EC (R^2^ = 0.235, *p* < 0.01), AK (R^2^ = 0.219, *p* < 0.01), NH_4_^+^-N (R^2^ = 0.194, *p* < 0.05), and TK (R^2^ = 0.132, *p* < 0.05). For fungi, the dominant driving factors included pH (R^2^ = 0.275, *p* < 0.01), AK (R^2^ = 0.224, *p* < 0.01), NH_4_^+^-N (R^2^ = 0.16, *p* < 0.05), and SWC (R^2^ = 0.152, *p* < 0.05) (Table S5).

Mantel analysis was performed to identify the key soil physicochemical variables affecting the diversity and richness of microbial communities (Figure 6B). For the bacterial community, observed ASV species correlated with TP, NH_4_^+^-N, and EC; diversity correlated with TN and TK; richness correlated with TP, TK, NH_4_^+^-N, and EC (Figure 6B(a)). For the fungal community, observed ASV species correlated with NO_3_^−^-N, AK, EC, TS, and pH; diversity correlated with NO_3_^−^-N, AK, and pH; richness correlated with NO_3_^−^-N, AK, TS, and pH (Figure 6B(b)). Overall, the key factors regulating the bacterial and fungal communities exhibited distinct differences, with fungal α-diversity being regulated by more key factors than bacterial α-diversity.

VPA demonstrated the explanatory power and contribution of soil properties and nutrients to the structural distribution of soil microbial communities (Figure 6C). For bacteria, soil nutrients (38.78%) > interaction of soil properties and nutrients (25.68%) > soil properties (15.13%) (Figure 6C(a)). For fungi, soil properties (34.06%) > interaction (28.25%) > soil nutrients (25.32%) (Figure 6C(b)). These results indicated that bacterial communities were more dependent on soil nutrients, whereas fungal communities were more dependent on soil properties.

### 2.4. Soil Quality Assessment

**Figure 7:**
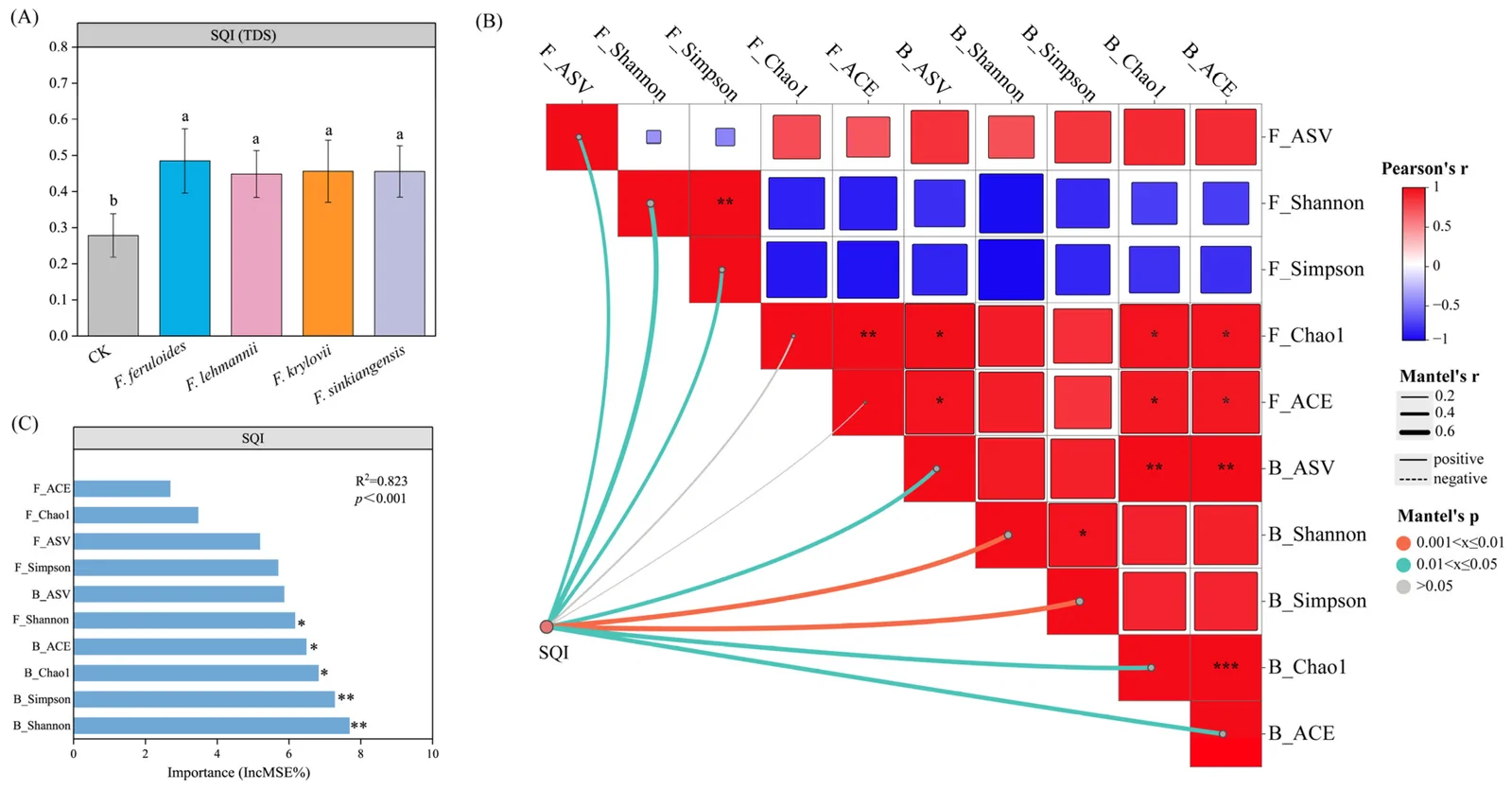
SQI (TDS) evaluation and key influencing factors. (**A**) SQIs of different *Ferula* species and growth stages (different lowercase letters indicate significant differences; *p* < 0.05). (**B**) Pearson correlation analysis of soil factors and the Mantel test for the SQI (* *p* < 0.05; ** *p* < 0.01; *** *p* < 0.001). (**C**) Random forest model analysis of key soil microbial factors associated with the SQI.

Soil quality index (SQI) based on 15 soil-related indicators showed EX had a significantly higher SQI than CK (*p* < 0.05). Specifically, the order of SQI increments among different *Ferula* species was as follows: *F. feruloides* (0.207) > *F. krylovii* (0.178) > *F. sinkiangensis* (0.177) > *F. lehmannii* (0.170) (Figure 7A).

Mantel test analysis showed that the SQI was significantly positively correlated with B_Shannon, B_Simpson, B_Chao1, B_ACE, B_ASV, F_Shannon, F_Simpson, and F_ASV (Figure 7B). The random forest model further identified that B_Shannon, B_Simpson, B_Chao1, B_ACE, and F_Shannon were the key variables affecting the SQI (*p* < 0.05) (Figure 7C).

## 3. Discussion

### 3.1. Regulatory Effects of Ferula Plants on Soil Biotic and Abiotic Factors

Plant growth alters the soil microenvironment through root exudation and residue decomposition, affecting physicochemical properties and microbial diversity, consistent with studies on desert plants and farmland crops. In the present study, *Ferula* cultivation significantly increased TN, TP, TK, NO_3_^−^-N, AP, AK, and SWC (especially SWC, TK, TP, AP, and AK) (Figure 1A), aligning with the finding by Chen et al. [27] that ephemeral plants drive soil properties in arid/semi-arid regions in early spring. Roots affect soil physicochemical properties via growth, distribution, metabolism, death, and residue decomposition; root exudates trigger rhizosphere effects to promote microbial activity and nutrient transformation, further modifying rhizosphere microenvironment.

*Ferula* cultivation significantly altered soil physicochemical properties and microbial diversity, with interspecific specificity and temporal dynamics (Figure 1C,D). This result is consistent with the finding by Gao et al. [28] that plant-driven changes in soil properties of different degraded grasslands are species-dependent. Higher microbial α-diversity in EX than CK may be attributed to rhizosphere effects from root exudates and litter. Specifically, as *Ferula* plants grow, litter-derived dissolved organic compounds regulate soil enzyme activity, promoting nutrient decomposition and providing substrates for microbial reproduction; root exudates (organic acids, amino acids, phenols, terpenoids, and volatile organic compounds supply carbon/nitrogen sources and energy [29]; litter decomposition supplements soil organic carbon pools. These factors together create favorable conditions for the enrichment of microbial communities, ultimately leading to an increase in soil microbial diversity. Additionally, improved soil nutrients (TP, TK, AP, and AK) and physicochemical parameters (SWC and pH) in EX provide a more suitable habitat for microbes. Notably, bacterial diversity was higher at the seedling stage (except *F. feruloides*), while fungal diversity peaked at the senescent stage, indicating distinct influence patterns on bacteria and fungi.

Different *Ferula* species showed significant differences in soil regulation (Figure S1): *F. sinkiangensis* had the highest AK and SWC, while *F. krylovii* had richer SOM and TN, possibly due to differences in root morphology, exudate composition, nitrogen fixation, and nutrient utilization strategies. Root exudates can directly input organic nitrogen substrates into the soil. Additionally, they can modulate the community composition and activity of nitrogen-fixing bacteria to promote biological nitrogen fixation, which further facilitates the accumulation of total soil nitrogen. Meanwhile, the growth stage of *Ferula* exerted significant effects on soil properties, with most soil indicators peaking at the senescent stage (Figure 1D), as increased root exudation and litter return promote nutrient accumulation (e.g., SOM, TN) and microbial activity enhances nutrient transformation. Cluster analysis further showed soil properties at the seedling stage were distinct from those at the mature and senescent stages (except *F. feruloides*), reflecting staged shaping of soil environments by plant resource allocation strategies. This discovery provides a scientific reference for the restoration and utilization of soil ecosystems in nutrient-deficient arid and semi-arid regions.

### 3.2. Remodeling Effects of Ferula Plants on Soil Microbial Communities

*Ferula* cultivation increased the numbers of bacterial/fungal ASVs and shared ASVs (Figure 2A), indicating root “filtering effects” enriched more microorganisms with common functions, which is consistent with the rhizosphere effect theory proposed by Peiffer et al. [30]. Changes in microbial community structure are the core manifestation of plant regulation on soil ecosystems [31]. NMDS and PCoA confirmed *Ferula* significantly remodeled microbial community structure (Figure 2B–E), with species and growth stage driving differentiation; bacteria were more sensitive (61.82% variation explained) than fungi (41.8%), possibly due to faster bacterial reproduction and stronger adaptability to environmental changes.

The dominant bacterial phyla (including Pseudomonadales and Acidobacteria) and the fungal phyla (including Ascomycota and Mortierellales) were enriched in EX (Figure 3A–D). These taxa, together with other microbial communities, participate in the soil nutrient cycling processes (organic carbon decomposition and nitrogen transformation); the increased abundance of these microbial communities contributes to enhancing soil fertility [32], which may suggest that cultivating Felugras plants can enhance soil ecological functions. Ascomycota (dominant in all samples) has strong organic matter decomposition ability and environmental adaptability, promoting carbon cycling via extracellular enzyme secretion [33], which may benefit *Ferula* growth and adaptation. Notably, fungal genus Tulostoma was only detected in CK (Figure 3D), possibly due to *Ferula*-induced changes in rhizosphere physicochemical properties (mismatching Tulostoma growth requirements), intensified interspecific competition from rhizosphere fungal community remodeling [34]. According to the results of this study, cultivation of *Ferula* plants facilitated the enrichment of Ascomycota, which emerged as the core responsive taxa in the rhizosphere fungal community. These enriched fungi vied with Tulostoma for soil nutrients and survival niches, thus undermining the dominant status of Basidiomycota—the phylum to which Tulostoma belongs. In addition, Tulostoma mycelial growth/spore germination may be selectively inhibited by *Ferula* root exudates (phenolic acids, terpenoids) [35], which is also an important reason for the decrease in its abundance in rhizosphere soil, although the direct regulatory effect on microbial communities needs further study. LEfSe-identified biomarkers (e.g., Pseudomonadota, *Verticillium*, and *Solicoccozyma* in EX) may be unique functional taxa in the rhizosphere of *Ferula*, providing targets for screening soil-improving microorganisms.

Co-occurrence networks are effective tools for reflecting dominant microbial flora and their interactions, and the relationships between these microorganisms are crucial for maintaining the structure of microbial communities in various environments [36,37]. Co-occurrence networks showed more taxa in EX than in CK (Figure 4, Table S4), indicating *Ferula* cultivation is conducive to the formation of more connections between soil microbial taxa, making the network closer [38,39,40]. Higher nodes/edges and average degree in EX is consistent with the finding by Morriën et al. [37] that vegetation restoration can alter the co-occurrence structure of microbial communities. Lower average clustering coefficient in EX suggests *Ferula* may promote the functional differentiation of soil microbial taxa, and different taxa perform their respective duties to form a more efficient collaborative system rather than simply dense connections [41,42].

Changes in microbial functions reflect soil ecosystem service capacity. In this study, higher bacterial Metabolism and Environmental Information Processing gene abundances in EX (Figure 5A,B) indicate enhanced microbial activity in nutrient transformation; increased fungal Pathotroph-Saprotroph-Symbiotroph and Symbiotroph proportions (Figure 5C,D) promote plant nutrient absorption and stress resistance [43]. These changes suggest *Ferula* plants optimize material cycling and energy flow of soil ecosystems by reshaping microbial functions.

### 3.3. Association Mechanisms Between Environmental Factors and Microbial Communities

Environmental factors drive microbial community dynamics, which are central to ecosystem regulation. db-RDA showed EC, AK, and NH_4_^+^-N drive bacterial communities, while pH, AK, and SWC dominate fungal communities (Figure 6A). This is related to the differences in the adaptability of different soil microbial taxa to environmental factors, which is consistent with the finding by Wang and Tang [44] that bacteria are sensitive to salinity (EC) and available nutrients (AK, NH_4_^+^-N), while fungi are more susceptible to the regulation of soil pH and moisture conditions. Mantel analysis confirmed bacterial diversity correlates with total nutrients (TP, TN), while fungal diversity is affected by available nutrients (NO_3_^−^-N, AK) and pH (Figure 6B), indicating differentiated nutrient utilization strategies between bacteria and fungi.

Given that microbial community dynamics are driven by multiple factors, and the assembly of root-associated microbiomes is comprehensively affected by soil conditions, it becomes difficult to ascertain the relative contribution of a single soil factor to the full process of microbial succession [45,46]. VPA showed soil nutrients explain more bacterial variation (38.78%), while soil properties drive fungal variation (34.06%) (Figure 6C), which is consistent with the finding by Duan et al. [47] that abiotic factors affect different microbial taxa are “heterogeneously”. The relatively low explanation rates indicate that in addition to soil abiotic factors, biotic factors such as root exudates of *Ferula* plants and rhizosphere interactions are also key drivers governing the assembly of microbial communities. This may stem from distinct nutrient acquisition strategies: bacteria depend on directly available nutrients, while fungi utilize complex organic substances via hyphal networks [48].

### 3.4. Potential of Ferula Plants in Ameliorating Soil Quality

Soil quality can reflect the comprehensive capacity of ecosystems to maintain biodiversity, facilitate nutrient cycling and enhance plant productivity. Arid soil is sensitive to environmental changes, with plants as key drivers of soil property changes. TDS-based SQI evaluation showed *Ferula* cultivation significantly improved soil quality (Figure 7A), which is consistent with the finding by Jiang et al. [49] and Li et al. [50] that plants are key to soil improvement. This directly verified the value of *Ferula* plants in improving soil quality in arid regions, which is of great practical significance for the restoration of soil ecological environments in arid areas. Different plant species exert varying effects on soil quality [17,18]. *F. feruloides* showed the best improvement effect, attributed to comprehensive advantages in rhizosphere nutrients and microbial diversity.

Mantel test analysis and Random forest identified key factors (B_Shannon, B_Simpson, B_Chao1, B_ACE, and F_Shannon) (Figure 7B,C) are microbial community drivers; their synergistic effects promote soil quality improvement. This finding provides clear target indicators for enhancing the SQI in arid regions, suggesting that optimization of soil improvement effects can be achieved by regulating these indicators. This indicates that *Ferula* improves soil quality mainly by optimizing nutrient status and physicochemical properties, with microbial community optimization consolidating effects. This aligns with soil quality improvement mechanisms in agricultural ecosystems [51,52], providing an important basis for screening *Ferula* species for ecological restoration of degraded soil remediation in arid regions.

In conclusion, *Ferula* cultivation positively improves soil ecosystems and quality in arid regions. Bacterial communities are more sensitive to *Ferula* cultivation, while fungal communities are more affected by soil physicochemical factors. *Ferula* enhances the SQI by regulating physicochemical properties and microbial communities, with *F. feruloides* as the optimal species. Further research is needed to characterize the mechanisms of *Ferula* plant traits and metabolites in soil quality regulation, but this study provides valuable insights into plant−soil quality−microbial community interactions in arid regions.

## 4. Materials and Methods

### 4.1. Research Area and Sample Collection

The study was conducted in the suburban area of Shihezi City, Xinjiang (44°15′50″ N, 86°03′31″ E), at 490 m a.s.l., characterized by a temperate continental arid climate and sandy soil. Artificial sowing of *Ferula* was carried out in September 2020 using wild-collected seeds (provided by Xinjiang Meinong *Ferula* Co., Ltd.) on an abandoned wasteland. Before sowing, weeds and impurities were removed, with 5 cm-deep shallow furrows; row and plant spacing were 30 cm and 40 cm, respectively, and 10 m isolation belts separated species to avoid interference. No fertilizers/pesticides were used, with regular manual weeding to simulate natural conditions.

In the *Ferula* planting area (EX), a completely randomized block design was employed to establish 12 independent quadrats (4 *Ferula* species × 3 replicates; 5 m × 5 m), with a spacing of no less than 5 m between quadrats to avoid mutual interference. Soil samples from adjacent blank plots without *Ferula* planting were collected as controls (CK) (3 replicates), with synchronized sampling timing consistent with each growth stage. Sampling depth and processing methods were also kept consistent with those used for *Ferula* root-soil samples to ensure the validity of comparative analysis. In this study, root-soil samples of four *Ferula* species (*F. feruloides*, *F. lehmannii*, *F. krylovii*, and *F. sinkiangensis*) were collected at three key growth stages: late March 2024 (seedling stage, when plant height reached approximately 2 cm), early May 2024 (mature stage, when mature *Ferula* plants were in full bloom), and late June 2024 (senescent stage, when aboveground plant parts had completely withered). The rhizosphere soil sampling method used in this study is destructive, making it impossible to repeatedly collect samples from the same marked plant during consecutive growth stages. Therefore, under identical microhabitat conditions, independent *Ferula* plants with uniform growth were selected, and samples were collected at each distinct growth stage. Each plot represents one experimental replicate, and composite rhizosphere soil samples collected within a plot were treated as one replicate sample. Within each plot, 3 well-growing *Ferula* plants were selected using the “Z”-shaped sampling method. A sterilized small shovel was used to carefully excavate around the roots (to avoid damage) and peel off rhizosphere soil (depth: 0–40 cm). Impurities (stones with a diameter of >2 cm, plant residues, plastic films) were removed, and soil samples from 3 plants in the same plot were thoroughly mixed to prepare a composite soil sample. Each composite sample was divided into three subsamples: ① immediately placed into a pre-weighed aluminum box for soil water content determination; ② quickly aliquoted into sterilized 15 mL centrifuge tubes and stored in liquid nitrogen for microbial community analysis; ③ stored in self-sealing bags and transported to the laboratory for physicochemical property determination.

### 4.2. Determination of Soil Physicochemical Properties

Rhizosphere soil samples were air-dried indoors with occasional turning for uniformity, then sieved through a 2 mm nylon sieve [53]. Soil pH was measured with a pH meter in a 1:2.5 soil−water suspension. Soil water content (SWC) was determined gravimetrically. Soil organic matter (SOM) was analyzed via the potassium dichromate volumetric−external heating method. Total nitrogen (TN) was measured after perchloric acid-sulfuric acid digestion; total phosphorus (TP) was determined by acid digestion—molybdenum antimony resistance colorimetry. Total potassium (TK) and available potassium (AK) were detected using atomic absorption spectrophotometry. Nitrate nitrogen (NO_3_^−^-N) and ammonium nitrogen (NH_4_^+^-N) were extracted with 0.01 M CaCl_2_ and determined colorimetrically. Available phosphorus (AP) was analyzed by the sodium bicarbonate extraction−molybdenum antimony resistance colorimetry. Electrical conductivity (EC) and total salt (TS) were measured with a conductivity meter and the dry residue method, respectively.

### 4.3. Amplicon Sequencing of Soil Microorganisms

Soil genomic DNA was extracted via the CTAB method, quantified with a NanoDrop 2000 (Thermo Fisher Scientific, Waltham, MA, USA) and diluted to 1 ng/μL with sterile water. PCR amplification was performed using barcode-specific primers [54], Phusion^®^ High-Fidelity PCR Master Mix (New England Biolabs, Ipswich, MA, USA) and high-fidelity enzymes were as follows: 16S V4 region primers (515F/806R) for bacteria and ITS1 region primers (ITS5-1737F/ITS2-2043R) for fungi. PCR conditions were as follows: pre-denaturation at 95 °C for 3 min; 32 cycles of 95 °C/30 s, 52 °C/30 s, 72 °C/30 s; final extension at 72 °C for 5 min; 4 °C hold. PCR products were verified by 2% agarose gel electrophoresis, recovered with AxyPrep Kit (Axygen, Union City, CA, USA), quantified via Quantus™ Fluorometer (Promega, Promega Corporation, Madison, WI, USA) and sequenced on NovaSeq 6000 after library construction (Illumina kit, San Diego, CA, USA) and qualification.

### 4.4. Data Processing and Statistical Analysis

To enhance the accuracy and reliability of the information analysis results, the raw sequencing data obtained from Illumina NovaSeq platforms underwent quality control and assembly to generate clean data [55]; the clean data were then subjected to chimeric sequence filtering to obtain effective data suitable for subsequent analyses. The Deblur tool was employed to perform noise reduction on this effective data [56]; Deblur compared the Hamming distances between sequences within a sample and those between different samples against an upper-bound error curve, and combined this approach with a greedy algorithm to identify Amplicon Sequence Variants (ASVs). Deblur was implemented using QIIME 2. Based on the ASV analysis results, on the one hand, we performed species annotation for each ASV sequence to obtain the corresponding species information and species-based abundance distribution profiles. At the same time, we conducted ASV abundance calculation, alpha diversity analysis, and Venn diagram analysis to clarify the species richness and evenness within each sample. On the other hand, we applied dimensionality reduction methods including PCoA and NMDS to explore the differences in community structure among different samples or groups. The raw sequencing reads of bacteria and fungi have been deposited in NCBI, with their corresponding BioProject accession numbers being PRJNA1397348 (https://dataview.ncbi.nlm.nih.gov/object/PRJNA1397348?reviewer=t9laf5h08n9lvg5qsrivsp6lhd) (accessed on 17 August 2026) and PRJNA1397366 (https://dataview.ncbi.nlm.nih.gov/object/PRJNA1397366?reviewer=untpicrtg5ltg7e5f8bg9pumo0) (accessed on 17 August 2026), respectively.

MAFFT (v7.520) was used for multiple sequence alignment [7], with normalization by the smallest sample size. Alpha diversity indices were calculated via R (v4.2.0) phyloseq (v 1.40.0) and vegan (v 2.6.2); PCoA/NMDS and LEfSe (LDA = 4) were performed for ordination and biomarker analysis. The phyloseq (v1.40.0) package in R software (v4.2.0) was used to calculate Unifrac distances; PCoA and NMDS plots were generated using R software (v4.2.0).

The top 100 microbial genera were selected based on relative abundance for correlation analysis, after which the following filtering criteria were applied: (1) remove connections with an absolute correlation coefficient of ≤0.8; (2) filter out node self-connections; (3) remove connections with node abundance less than 0.005%. The calculation of species correlation was implemented using FastSpar (v 1.0.0), visualized with Cytoscape 3.7.2, and topological parameters/modularity (MCODE) analyzed. VPA and Mantel tests were conducted via R vegan, ggcor, and ggplot2 packages to quantify environmental factor explanatory power.

Soil quality index (SQI) was calculated based on 15 indicators: ① construct the Total Data Set (TDS); ② convert indicators to linear scores (0–1) using Formula (1) [57]; ③ calculate indicator weights via PCA (communal variance) (Table S6); ④ compute the SQI with Formula (2).

(1) $$\mathrm{SL}=\frac{x\;-\;L}{H-L}\;$$

where SL is the linear score, x is the measured value, L is the minimum value, and H is the maximum value.

(2) $$\mathrm{SQI}=\sum_{i=1}^{n}\mathrm{Wi}\;\times \;\mathrm{si}$$

where Si is the score of the i-th indicator, n is the number of indicators, and Wi is the weight of the i-th indicator [58].

An independent-samples *t*-test was also performed to compare the *Ferula*-planted (EX) and unplanted (CK) groups using IBM SPSS Statistics 25 for statistical analysis. The Shapiro−Wilk test was employed to assess data normality, and a two-sided *p* value of < 0.05 was considered statistically significant. A two-way ANOVA of soil physicochemical properties and microbial community diversity indices was conducted using IBM SPSS Statistics 25, with Bonferroni’s post-hoc test used for multiple comparisons, and the significance threshold was set at *p* < 0.05.

For taxon-level comparison, one-way ANOVA was performed on taxonomic relative abundances across groups. To reduce false-positive risks caused by extensive multiple testing, raw *p*-values were adjusted via the Benjamini−Hochberg false-discovery rate (FDR) approach to produce q-values. Considering the compositional and sparse nature of amplicon sequencing data, ANCOM-BC was additionally applied as a compositional-data-aware differential-abundance test. *p*-values reaching software computational limits are reported as *p* < 2.2 × 10^−16^ rather than *p* = 0. Taxa with FDR-adjusted q-values of < 0.05 were considered significantly different among groups.

Random Forest analysis was conducted with the “rfPermute” package in R (parameters: set.seed (123); ntree = 500; nperm = 1000; nrep = 1000), and variable importance scores (% IncMSE) were predicted with the “importance” function. Then, all results were visualized (including principal component analysis, box plots, stacked graphs, and bar charts) using the Origin 2021 software.

## 5. Conclusions

This study systematically analyzed the effects of ephemeroid *Ferula* plants on soil physicochemical properties and microbial communities in arid regions, and comprehensively evaluated soil quality. The results showed *Ferula* cultivation significantly affected soil biotic and abiotic factors (*p* < 0.05), with interspecific specificity and temporal dynamics. Changes in soil physicochemical properties were associated with rhizosphere microbial community differences: bacteria showed closer associations with nutrients, while fungi exhibited stronger links to soil properties. *Ferula* significantly improved the arid SQI, with *F. feruloides* identified as the optimal species. There is a significant correlation between the rhizosphere bacterial diversity index and soil quality. This study reveals the chain-like relationships of *Ferula* cultivation on the “soil environment−microbial community−soil quality” continuum, clarifies the core roles of interspecific specificity and temporal dynamics, and enriches the theoretical system of plant−soil−microbe interactions. It provides suitable plant resources and technical references for degraded soil remediation in arid regions, with important ecological and application value.

## Data Availability

The raw sequencing data used in this study were deposited in the NCBI Sequence Read Archive under BioProject accession number PRJNA1397348 (https://dataview.ncbi.nlm.nih.gov/object/PRJNA1397348?reviewer=t9laf5h08n9lvg5qsrivsp6lhd) (accessed on 17 August 2026) and PRJNA1397366 (https://dataview.ncbi.nlm.nih.gov/object/PRJNA1397366?reviewer=untpicrtg5ltg7e5f8bg9pumo0) (accessed on 17 August 2026). All data generated or analyzed during this study are included in this published article.

## Supplementary Materials

The following supporting information can be downloaded at: https://www.mdpi.com/article/10.3390/plants15182846/s1, Figure S1: Comparison of soil factors among different *Ferula* species. Different lowercase letters indicate significant differences (*p* < 0.05). Abbreviations: SOM (Soil organic matter), TN (Total nitrogen), TP (Total phosphorus), TK (Total potassium), SAK (Available potassium), NO_3_^−^-N (Nitrate nitrogen), NH^+^-N (Ammonium nitrogen), AP (Available phosphorus), EC (Electrical conductivity), TS (Total salt), SWC (Soil water content); F_ASV, F_Shannon, F_Simpson, F_Chao1, F_ACE (Soil fungal diversity indices); B_ASV, B_Shannon, B_Simpson, B_Chao1, B_ACE (Soil bacterial diversity indices). Figure S2. Bar charts of LDA value distribution based on ASV, showing the species with significant differences in abundance among different groups. The first uppercase letters M, C, S represent the seedling stage, mature stage, and senescent stage, respectively. The second uppercase letters X, D, G, T refer to *F. sinkiangensis*, *F. feruloides*, *F. lehmannii*, and *F. krylovii*, respectively. For instance, MD denotes *F. feruloides* at the mature stage; Table S1: Effects of treatment and stage on rhizosphere soil physicochemical properties and microbial diversity; Table S2: Effects of cultivation *Ferula* and growth stages on rhizosphere soil physicochemical properties and microbial diversity; Table S3: Effects of *Ferula* species and growth stages on the community composition abundance of bacteria and fungi; Table S4: The number of bacterial and fungal taxa at the phylum level in the microbial networks in CK and EX; Table S5: Results for db-RDA testing the effects of soil physicochemical properties on the composition and distribution of the soil microbial community; Table S6. Principal component analysis of soil physicochemical factors in different groups.

## Abbreviations

The following abbreviations are used in this manuscript:

AK	available potassium
AP	available phosphorus
ASVs	Amplicon Sequence Variants
db-RDA	distance-based redundancy analysis
EC	electrical conductivity
KEGG	Kyoto Encyclopedia of Genes and Genomes
LEfSe	Linear Discriminant Analysis Effect Size
LDA	Linear Discriminant Analysis
NH_4_^+^-N	ammonium nitrogen
NMDS	non-metric multidimensional scaling
NO_3_^−^-N	nitrate nitrogen
PCA	principal component analysis
PCoA	Principal Coordinates Analysis
SOM	soil organic matter
SWC	soil water content
TK	total potassium
TN	total nitrogen
TP	total phosphorus
TS	total salt
SQI	soil quality index
VPA	Variance Partitioning Analysis
