# Supplementary material for: Dynamic Changes in Rhizosphere Microecology of Ephemeroid Ferula and Their Effects on Soil Quality in Arid Regions
Source: Plants (Basel). 2026 Sep 17;15(18):2846. doi: 10.3390/plants15182846 (PMC13610510; doi:10.3390/plants15182846)
Supplement: Supplementary file 1 [file plants-15-02846-s001.zip › plants-4549388-supplementary.pdf]

Dynamic changes in rhizosphere microecology of ephemeroid *Ferula* and their effects on soil quality in arid regions

Hanli Dang [1], Zhilin Zhang [1], Aifang Ma [1], and Tao Zhang [1*]

1 Ministry Key Laboratory of Xinjiang Phytomedicine Resource and Utilization, Key Laboratory of Oasis Town and Mountain-Basin System Ecology of Bingtuan, College of Life Sciences, Shihezi University, Shihezi 832003, China

* Correspondence and requests for materials should be addressed to Tao Zhang (E-mail: zhangtao@shzu.edu.cn).

Additional file

Figure S1. Comparison of soil factors among different *Ferula* species.

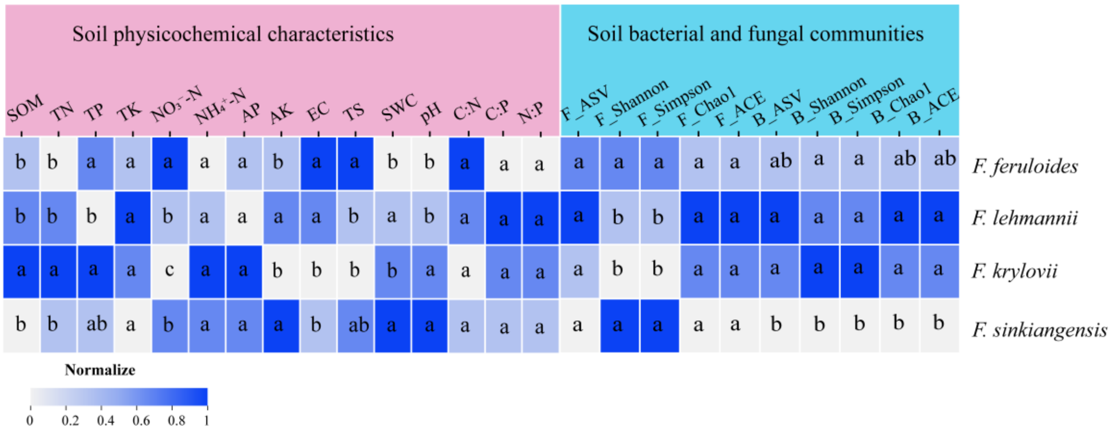

Figure S1. Comparison of soil factors among different *Ferula* species. Different lowercase letters indicate significant differences ($p < 0.05$). Abbreviations: SOM (Soil organic matter), TN (Total nitrogen), TP (Total phosphorus), TK (Total potassium), AK (Available potassium), NO_3^--N (Nitrate nitrogen), NH_4^+-N (Ammonium nitrogen),

AP (Available phosphorus), EC (Electrical conductivity), TS (Total salt), SWC (Soil water content); F_ASV, F_Shannon, F_Simpson, F_Chao1, F_ACE (Soil fungal diversity indices); B_ASV, B_Shannon, B_Simpson, B_Chao1, B_ACE (Soil bacterial diversity indices).

Figure S2. Bar charts of LDA value distribution based on ASV.

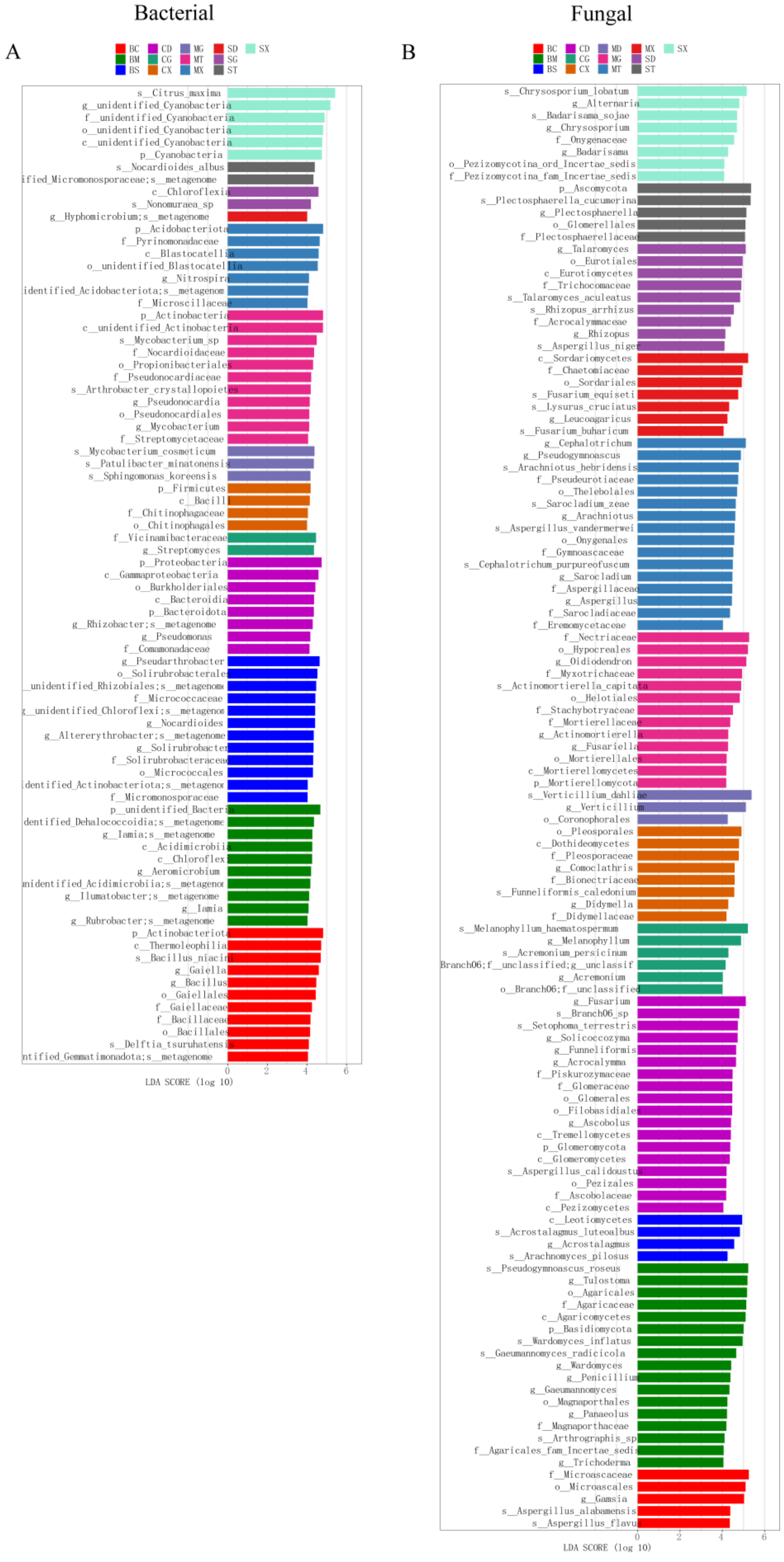

Figure S2. Bar charts of LDA value distribution based on ASV, showing the species with significant differences in abundance among different groups. The first uppercase letters M, C, S represent the seedling stage, mature stage, and senescent stage, respectively. The second uppercase letters X, D, G, T refer to *F. sinkiangensis*, *F. feruloide*s, *F. lehmannii*, and *F. krylovii*, respectively. For instance, MD denotes *F. feruloide*s at the mature stage.

Supplementary Table 1-6

Table S1. Effects of treatment and stage on rhizosphere soil physicochemical properties and microbial diversity

Source	Dependent variable	Type III Sum of Squares	Degrees of freedom	Mean Square	F	*p* value
treatment	SOM	0.134	1	0.134	0.248	0.627
	TN	0.001	1	0.001	1.209	0.293
	TP	0.003	1	0.003	7.08	0.021
	TK	0.579	1	0.579	5.002	0.045
	NO_3^--N	2.905	1	2.905	0.121	0.734
	NH_4^+-N	0.012	1	0.012	0.02	0.891
	AP	5.513	1	5.513	6.091	0.03
	AK	9546.534	1	9546.534	85.108	0
	EC	496.125	1	496.125	5.027	0.045
	TS	0.002	1	0.002	0.083	0.778
	SWC	7.379	1	7.379	26.766	0
	pH	0.006	1	0.006	20.213	0.001
	F_ASV	4449.389	1	4449.389	14.42	0.003
	F_Shannon	0.901	1	0.901	6.682	0.024
	F_Simpson	0.006	1	0.006	2.559	0.136
	F_Chao1	375.554	1	375.554	0.662	0.432
	F_ACE	1502.543	1	1502.543	2.747	0.123
	B_ASV	25481.531	1	25481.531	3.818	0.074
	B_Shannon	0.079	1	0.079	8.376	0.013
	B_Simpson	0.000	1	0.00005339	11.44	0.005
	B_ACE	33006.256	1	33006.256	3.711	0.078
	B_Chao1	31543.017	1	31543.017	3.042	0.107
stage	SOM	14.497	2	7.248	13.435	0.001
	TN	0.004	2	0.002	1.863	0.197
	TP	0.002	2	0.001	2.354	0.137
	TK	0.488	2	0.244	2.106	0.164
	NO_3^--N	1564.400	2	782.2	32.572	0
	NH_4^+-N	16.580	2	8.29	14.001	0.001
	AP	1.610	2	0.805	0.889	0.436
	AK	4142.747	2	2071.374	18.466	0
	EC	2984.632	2	1492.316	15.121	0.001
	TS	0.141	2	0.07	2.833	0.098
	SWC	119.386	2	59.693	216.518	0
	pH	0.029	2	0.015	48.628	0
	F_ASV	6980.632	2	3490.316	11.312	0.002

	F_Shannon	1.516	2	0.758	5.623	0.019
	F_Simpson	0.013	2	0.007	2.962	0.09
	F_Chao1	12235.868	2	6117.934	10.792	0.002
	F_ACE	11617.350	2	5808.675	10.619	0.002
	B_ASV	114825.444	2	57412.722	8.602	0.005
	B_Shannon	0.158	2	0.079	8.409	0.005
	B_Simpson	0.000	2	0.00002022	4.333	0.038
	B_ACE	132706.292	2	66353.146	7.459	0.008
	B_Chao1	139630.651	2	69815.325	6.733	0.011
treatment * stage	SOM	2.840	2	1.42	2.632	0.113
	TN	0.008	2	0.004	3.985	0.047
	TP	0.008	2	0.004	8.888	0.004
	TK	0.311	2	0.155	1.341	0.298
	NO_3^--N	3502.882	2	1751.441	72.932	0
	NH_4^+-N	1.229	2	0.615	1.038	0.384
	AP	1.702	2	0.851	0.94	0.418
	AK	2539.764	2	1269.882	11.321	0.002
	EC	13297.021	2	6648.51	67.367	0
	TS	0.228	2	0.114	4.588	0.033
	SWC	17.485	2	8.743	31.712	0
	pH	0.201	2	0.1	331.739	0
	F_ASV	1771.132	2	885.566	2.87	0.096
	F_Shannon	0.406	2	0.203	1.504	0.261
	F_Simpson	0.007	2	0.003	1.553	0.251
	F_Chao1	3621.576	2	1810.788	3.194	0.077
	F_ACE	2976.255	2	1488.127	2.72	0.106
	B_ASV	38943.000	2	19471.5	2.917	0.093
	B_Shannon	0.018	2	0.009	0.963	0.409
	B_Simpson	0.000	2	0.000008222	1.762	0.213
	B_ACE	47667.927	2	23833.964	2.679	0.109
	B_Chao1	48818.410	2	24409.205	2.354	0.137

$p < 0.05$ indicates statistical significance.

Table S2. Effects of cultivation *Ferula* and growth stages on rhizosphere soil physicochemical properties and microbial diversity

Source	Dependent variable	Type III Sum of Squares	Degrees of freedom	Mean Square	F	*p* value
Plant species	SOM	6.246	3	2.082	7.552	0.001
	TN	0.027	3	0.009	3.454	0.032
	TP	0.043	3	0.014	4.259	0.015
	TK	0.069	3	0.023	0.137	0.937
	NN	5186.606	3	1728.869	17.89	0
	AN	7.06	3	2.353	1.673	0.199
	AP	8.416	3	2.805	1.055	0.386
	AK	3138.19	3	1046.063	5.672	0.004
	EC	13718.333	3	4572.778	12.954	0
	TS	0.224	3	0.075	2.886	0.057
	SWC	8.484	3	2.828	6.578	0.002
	pH	0.018	3	0.006	19.357	0
	C:N	10.993	3	3.664	0.942	0.436
	C:P	20.959	3	6.986	2.556	0.079
	N:P	0.033	3	0.011	1.742	0.185
	F_ASV	396.222	3	132.074	0.444	0.724
	F_Shannon	0.44	3	0.147	7.148	0.001
	F_Simpson	0.002	3	0.001	5.173	0.007
	F_Chao1	3844.831	3	1281.61	1.18	0.338
	F_ACE	5292.319	3	1764.106	1.66	0.202
	B_ASV	148812.75	3	49604.25	3.346	0.036
	B_Shannon	0.741	3	0.247	6.328	0.003
	B_Simpson	0	3	0	5.01	0.008
	B_Chao1	169343.669	3	56447.89	3.271	0.039
	B_ACE	160096.493	3	53365.498	3.247	0.04
growth period	SOM	12.743	2	6.371	23.112	0
	TN	0.02	2	0.01	3.944	0.033
	TP	0.005	2	0.002	0.699	0.507
	TK	0.548	2	0.274	1.623	0.218
	NN	9425.753	2	4712.877	48.768	0
	AN	51.488	2	25.744	18.305	0
	AP	12.096	2	6.048	2.275	0.125
	AK	10226.994	2	5113.497	27.726	0
	EC	21541.722	2	10770.861	30.512	0
	TS	0.192	2	0.096	3.709	0.039
	SWC	279.991	2	139.996	325.641	0
	pH	0.217	2	0.109	349.179	0
	C:N	51.028	2	25.514	6.558	0.005

	C:P	11.681	2	5.84	2.137	0.14
	N:P	0.019	2	0.009	1.51	0.241
	F_ASV	11079.056	2	5539.528	18.624	0
	F_Shannon	0.743	2	0.372	18.092	0
	F_Simpson	0.002	2	0.001	8.766	0.001
	F_Chao1	9910.244	2	4955.122	4.562	0.021
	F_ACE	9981.465	2	4990.732	4.695	0.019
	B_ASV	68960.889	2	34480.444	2.326	0.119
	B_Shannon	0.266	2	0.133	3.413	0.05
	B_Simpson	0	2	0	3.346	0.052
	B_Chao1	70819.93	2	35409.965	2.052	0.15
	B_ACE	71557.6	2	35778.8	2.177	0.135
Plant species * growth period	SOM	13.227	6	2.205	7.997	0
	TN	0.052	6	0.009	3.349	0.015
	TP	0.048	6	0.008	2.358	0.062
	TK	3.206	6	0.534	3.168	0.02
	NN	12604.851	6	2100.808	21.739	0
	AN	24.53	6	4.088	2.907	0.028
	AP	58.905	6	9.817	3.693	0.01
	AK	34123.635	6	5687.272	30.837	0
	EC	43547.833	6	7257.972	20.561	0
	TS	0.475	6	0.079	3.059	0.023
	SWC	15.872	6	2.645	6.153	0.001
	pH	0.093	6	0.016	50.036	0
	C:N	170.216	6	28.369	7.292	0
	C:P	72.196	6	12.033	4.403	0.004
	N:P	0.037	6	0.006	0.988	0.455
	F_ASV	5029.611	6	838.269	2.818	0.032
	F_Shannon	0.516	6	0.086	4.189	0.005
	F_Simpson	0.002	6	0	3.149	0.02
	F_Chao1	10905.5	6	1817.583	1.673	0.171
	F_ACE	9789.989	6	1631.665	1.535	0.21
	B_ASV	74321.333	6	12386.889	0.835	0.555
	B_Shannon	0.368	6	0.061	1.571	0.199
	B_Simpson	0	6	6.444E-5	2.086	0.093
	B_Chao1	82223.1	6	13703.85	0.794	0.584
	B_ACE	78141.869	6	13023.645	0.792	0.585

$p < 0.05$ indicates statistical significance.

Table S3. Effects of *Ferula* species and growth stages on the community composition abundance of bacteria and fungi.

**Bacterial phyta**	Proteobacteria		Acidobacteriota		Actinobacteriota		Actinobacteria		Gemmatimonadota		unidentified_Bacteria		Crenarchaeota		Cyanobacteria		Bacteroidota		Myxococcota	
	F	*p*	F	*p*	F	*p*	F	*p*	F	*p*	F	*p*	F	*p*	F	*p*	F	*p*	F	*p*
Species	2.148	0.121	6.95	**0.002**	8.582	**$<2.2\times10^{-16}$**	48.998	**$<2.2\times10^{-16}$**	0.778	0.518	3.886	**0.021**	2.206	0.113	4.42	**0.013**	1.416	0.262	6.31	**0.003**
Stage	2.282	0.124	0.708	0.503	20.873	**$<2.2\times10^{-16}$**	6.06	**0.007**	3.62	**0.042**	1.865	0.177	2.305	0.121	5.993	**0.008**	3.145	0.061	1.492	0.245
Species*Stage	5.109	**0.002**	2.035	**0.1**	4.762	**0.003**	2.334	0.064	0.465	0.827	1.081	0.401	0.307	0.927	2.325	0.065	1.575	0.198	1.904	0.121
**CK**	**0.146±0.007 b**		0.131±0.008 a		**0.210±0.006 a**		**0.160±0.011 a**		0.108±0.008 a		0.104±0.013 a		0.033±0.007 a		**0.001±0.000 b**		**0.011±0.003 b**		0.015±0.002 a	
**EX**	**0.176±0.005 a**		0.159±0.007 a		**0.135±0.005 b**		**0.126±0.006 b**		0.111±0.007 a		0.080±0.006 a		0.045±0.004 a		**0.043±0.008 a**		**0.025±0.003 a**		0.017±0.001 a	

**Bacterial genus**	*Nitrospira*		*Dongia*		*Streptomyces*		*Solirubrobacter*		*Bacillus*		*Nocardioides*		*Sphingomonas*		*Gaiella*		*Pseudarthrobacter*		unidentified_*Cyanobacteria*	
	F	*p*	F	*p*	F	*p*	F	*p*	F	*p*	F	*p*	F	*p*	F	*p*	F	*p*	F	*p*
Species	3.185	**0.042**	3.348	**0.036**	50.008	**$<2.2\times10^{-16}$**	13.241	**$<2.2\times10^{-16}$**	3.138	**0.044**	48.105	**0**	4.646	**0.011**	4.175	**0.016**	0.669	0.579	4.02	0.019
Stage	2.158	0.137	0.113	0.894	11.987	**$<2.2\times10^{-16}$**	14.277	**$<2.2\times10^{-16}$**	2.266	0.126	0.251	0.78	3.145	0.061	29.011	**$<2.2\times10^{-16}$**	2.61	0.094	5.353	0.012
Species*Stage	0.317	0.922	0.968	0.467	12.674	**$<2.2\times1$**	3.946	**0.007**	1.005	0.445	6.13	**0.001**	0.3	0.931	4.334	**0.004**	6.386	**$<2.2\times10^{-1}$**	2.356	0.062

			**0^{-16}**						**6**	
**CK**	0.005±0.001 a	0.007±0.001 a	0.011±0.001 a	**0.016±0.001 a**	0.010±0.003 a	**0.020±0.002 a**	0.015±0.001 a	**0.028±0.002 a**	0.027±0.005 a	**0.001±0.000 b**
**EX**	0.006±0.001 a	0.0087±0.000 a	0.010±0.001 a	**0.010±0.001 b**	0.013±0.002 a	**0.014±0.001 b**	0.015±0.001 a	**0.019±0.001 b**	0.027±0.002 a	**0.042±0.007 a**

**Fungal phyta**	Kickxellomycota		Olpidiomycota		Chytridiomycota		Aphelidiomycota		Mucoromycota		Glomeromycota		Mortierellomycota		Basidiomycota		Ascomycota	
	F	*p*	F	*p*	F	*p*	F	*p*	F	*p*	F	*p*	F	*p*	F	*p*	F	*p*
Species	13.055	< **2.2×10^{-16}**	5.645	**0.005**	2.333	0.099	187.133	< **2.2×10^{-16}**	34.934	< **2.2×10^{-16}**	22.738	< **2.2×10^{-16}**	9.21	< **2.2×10^{-16}**	10.602	< **2.2×10^{-16}**	18.468	< **2.2×10^{-16}**
Stage	20.048	< **2.2×10^{-16}**	0.631	0.541	0.846	0.441	194.974	< **2.2×10^{-16}**	47.776	< **2.2×10^{-16}**	54.308	< **2.2×10^{-16}**	0.031	0.97	4.713	**0.019**	5.968	**0.008**
Species*Stage	8.92	< **2.2×10^{-16}**	2.609	**0.043**	1.347	0.276	222.007	< **2.2×10^{-16}**	40.863	< **2.2×10^{-16}**	15.73	< **2.2×10^{-16}**	2.561	**0.046**	3.932	**0.007**	6.593	< **2.2×10^{-16}**
**CK**	**0.00002±0.000 b**		0.00044± 0.000 a		0.001 ±0.0001 a		0.00036±0.0001 a		0.004±0.001 a		**0.00032 ± 0.0001 b**		**0.011±0.003 b**		0.129±0.042 a		0.839±0.039 a	
**EX**	**0.00014±0.000 a**		0.0002±0.000 a		0.001±0.0001 a		0.002±0.001 a		0.002±0.001 a		**0.011 ±0.002 a**		**0.019±0.001 a**		0.062±0.008 a		0.885±0.01 a	

**Fungal genus**	*Aspergillus*		*Talaromyces*		*Verticillium*		*Solicoccozyma*		*Plectosphaerella*		*Oidiodendron*		*Pseudogymnoascus*		*Cephalotrichum*		*Fusarium*	
	F	*p*	F	*p*	F	*p*	F	*p*	F	*p*	F	*p*	F	*p*	F	*p*	F	*p*
Species	7.605	**0.001**	15.931	< **2.2×10^{-16}**	88.625	< **2.2×10^{-16}**	21.209	< **2.2×10^{-16}**	38.61	< **2.2×10^{-16}**	72.391	< **2.2×10^{-16}**	54.798	< **2.2×10^{-16}**	66.196	< **2.2×10^{-16}**	27.02	< **2.2×10^{-16}**
Stage	10.46	**0.001**	11.344	< **2.2×10^{-1}**	83.511	< **2.2×1**	11.617	< **2.2×10^{-16}**	44.726	< **$2.2\times10^{-}$**	43.465	< **2.2×10^{-16}**	16.354	< **2.2×10^{-16}**	65.769	< **2.2×1**	0.897	0.421

Species*Stage	5.563 **0.001**	15.28 **$< 2.2\times10^{-16}$**	158.255 **$< 2.2\times10^{-16}$**	16.238 **$< 2.2\times10^{-16}$**	16.182 **$< 2.2\times10^{-16}$**	65.277 **$< 2.2\times10^{-16}$**	30.817 **$< 2.2\times10^{-16}$**	25.655 **$< 2.2\times10^{-16}$**	15.219 **$< 2.2\times10^{-16}$**
**CK**	0.020±0.0103 a	0.015±0.011 a	**0.006±0.001 b**	**0.012±0.002 b**	**0.0002±0.0001 b**	0.039±0.019 a	0.047±0.007 a	0.119±0.037 a	0.052±0.016 a
**EX**	0.013±0.002 a	0.018±0.007 a	**0.024±0.006 a**	**0.023±0.002 a**	**0.027±0.008 a**	0.019±0.005 a	0.031±0.004 a	0.053±0.008 a	0.073±0.006 a

p < 0.05 indicates statistical significance.

Table S4. The number of bacterial and fungal taxa at the phylum level in the Microbial networks in CK and EX.

Networks	CK	number	EX	number
	unidentified_Bacteria	30	Proteobacteria	394
	Proteobacteria	11	Acidobacteriota	323
	Gemmatimonadota	9	Actinobacteria	292
	Actinobacteria	5	Actinobacteriota	276
	Crenarchaeota	5	unidentified_Bacteria	226
	Acidobacteriota	3	Gemmatimonadota	181
	Others	6	Myxococcota	87
			Bacteroidota	80
Bacterial networks			Firmicutes	37
			Verrucomicrobiota	33
			Methylomirabilota	20
			Crenarchaeota	17
			Planctomycetota	16
			Desulfobacterota	9
			Armatimonadota	6
			unidentified_Archaea	6
			Nitrospirota	4

			Bdellovibrionota	3
			Latescibacteria	2
			Cyanobacteria	1
			Sumerlaeota	1
			Kapabacteria	1
			Chlamydiae	1
			Others	142
Fungal networks	Ascomycota	18	Ascomycota	251
	Basidiomycota	9	Basidiomycota	33
	Glomeromycota	4	Mortierellomycota	10
	Mortierellomycota	2	Glomeromycota	7
	Mucoromycota	1	Chytridiomycota	4
	Others	1	Mucoromycota	4
			Aphelidiomycota	2
			Kickxellomycota	1
			Olpidiomycota	1
			Others	52

Table S5 Results for db-RDA testing the effects of soil physicochemical properties on the composition and distribution of soil microbial community

Bacterial community					Fungal community				
Variables	RDA1	RDA2	R^2	*p*-value	Variables	RDA1	RDA2	R^2	*p*-value
EC	-0.736	0.677	0.235	**0.004**	pH	-0.933	-0.361	0.275	**0.001**
AK	-1.000	-0.018	0.219	**0.005**	AK	-0.886	0.463	0.224	**0.005**
NH_4^+-N	-0.709	0.705	0.194	**0.015**	NH_4^+-N	-0.079	-0.997	0.160	**0.024**
TK	-0.967	0.255	0.132	**0.044**	SWC	0.864	-0.504	0.152	**0.032**
SOM	0.596	-0.803	0.109	0.096	TS	0.910	0.415	0.121	0.057
NO_3^--N	0.787	0.617	0.085	0.163	SOM	-0.972	0.234	0.092	0.149
pH	0.912	-0.410	0.078	0.184	EC	0.771	0.637	0.085	0.159
TS	-0.795	0.606	0.073	0.204	AP	-0.761	0.649	0.075	0.205
SWC	0.039	0.999	0.041	0.452	TK	-0.718	0.696	0.034	0.487
TP	-0.734	0.679	0.031	0.504	TN	-0.982	-0.186	0.030	0.527
AP	-0.998	0.055	0.021	0.622	NO_3^--N	0.666	0.746	0.022	0.628
TN	-0.665	-0.747	0.020	0.649	TP	-0.752	0.659	0.008	0.811

Description: R^2 is the determinant coefficients of the distribution of the soil microbial community by soil factors. *p* value is the *p*-value of significance test, and $p<$ 0.05 indicates statistical significance. Abbreviations: SOM, Soil organic matter; TN, Total nitrogen; TP, Total phosphorus; TK, Total potassium; SAK, available potassium; NO_3^--N, Nitrate nitrogen; NH_4^+-N, ammonium nitrogen; AP, Available phosphorus; AK, available potassium; EC, Electrical conductivity; TS, Total salt; SWC, Soil water content.

Table S6. Principal component analysis of soil physicochemical factors in different groups.

		principal component				Common factor variance	Weight	Membership degree
		PC1	PC2	PC3	PC4			
CK	SOM (g/kg)	0.631	0.731	-0.221	0.129	0.998	0.071	0.139
	TN (g/kg)	0.491	0.838	0.107	-0.186	0.989	0.070	0.296
	TP (g/kg)	-0.398	0.762	-0.424	-0.127	0.936	0.066	0.207
	C:N	0.464	0.070	-0.584	0.623	0.950	0.067	0.290
	C:P	0.874	0.404	-0.006	0.227	0.979	0.069	0.236
	N:P	0.760	0.445	0.381	-0.116	0.933	0.066	0.195
	TK (g/kg)	0.418	0.342	-0.314	-0.755	0.960	0.068	0.220
	NO_3-N (mg/kg)	-0.923	0.233	0.118	0.187	0.955	0.068	0.324
	NH_4-N(mg/kg)	-0.511	0.159	0.769	-0.064	0.882	0.062	0.286
	AP(mg/kg)	0.389	0.635	0.555	0.333	0.974	0.069	0.232
	AK(mg/kg)	0.929	0.061	0.029	0.088	0.875	0.062	0.270
	EC	-0.905	0.355	0.036	0.169	0.975	0.069	0.361
	TS (g/kg)	-0.552	0.776	0.103	-0.001	0.918	0.065	0.493
	SWC (%)	-0.732	0.427	-0.396	-0.021	0.875	0.062	0.264
	pH	0.845	-0.450	0.112	-0.071	0.934	0.066	0.373
	eigenvalue	7.030	3.901	1.921	1.281			
	Percentage of Variance (%)	46.866	26.008	12.805	8.543			
	Cumulative (%)	46.866	72.873	85.678	94.221			

		principal component				Common factor variance	Weight	Membership degree
		PC1	PC2	PC3	PC4			
F. feruloides	SOM (g/kg)	0.248	0.728	0.617	-0.118	0.985	0.070	0.523
	TN (g/kg)	-0.411	0.868	0.048	0.223	0.975	0.069	0.394
	TP (g/kg)	0.830	0.242	-0.393	-0.115	0.915	0.065	0.588
	C:N	0.703	-0.301	0.526	-0.343	0.980	0.070	0.543
	C:P	-0.198	0.554	0.804	-0.058	0.996	0.071	0.562
	N:P	-0.679	0.653	0.195	0.247	0.987	0.070	0.502
	TK (g/kg)	0.125	0.811	-0.482	-0.086	0.912	0.065	0.627
	NO_3-N (mg/kg)	0.911	-0.249	0.066	0.282	0.975	0.069	0.381
	NH_4-N(mg/kg)	-0.691	-0.579	0.286	0.264	0.965	0.069	0.369
	AP(mg/kg)	0.247	0.647	-0.193	0.437	0.708	0.050	0.567
	AK(mg/kg)	-0.698	0.409	-0.312	-0.482	0.984	0.070	0.530
	EC	0.950	-0.111	0.080	0.262	0.990	0.071	0.417
	TS (g/kg)	0.799	0.231	-0.017	0.261	0.760	0.054	0.438
	SWC (%)	-0.823	-0.342	-0.056	0.348	0.919	0.065	0.390
	pH	-0.962	-0.241	-0.038	0.067	0.990	0.071	0.464
	eigenvalue	6.932	4.046	1.963	1.102			
	Percentage of Variance (%)	46.212	26.974	13.086	7.344			
	Cumulative (%)	46.212	73.186	86.272	93.615			

		principal component					Common factor variance	Weight	Membership degree
		PC1	PC2	PC3	PC4	PC5			
F. lehmannii	SOM (g/kg)	0.564	-0.442	-0.524	0.283	-0.283	0.949	0.066	0.542
	TN (g/kg)	0.046	0.384	0.441	0.645	-0.482	0.991	0.069	0.408
	TP (g/kg)	-0.736	0.329	-0.422	0.406	0.055	0.997	0.069	0.731
	C:N	0.416	-0.581	-0.662	-0.177	0.082	0.986	0.069	0.662
	C:P	0.821	-0.439	0.147	-0.237	-0.185	0.980	0.068	0.198
	N:P	0.741	-0.184	0.561	-0.147	-0.270	0.992	0.069	0.233
	TK (g/kg)	-0.373	-0.578	0.119	0.528	0.353	0.890	0.062	0.337
	NO_3-N (mg/kg)	0.902	0.066	0.108	0.218	0.331	0.986	0.069	0.455
	NH_4-N(mg/kg)	0.108	0.867	-0.202	-0.174	0.384	0.981	0.068	0.514
	AP(mg/kg)	0.268	0.860	-0.155	-0.084	-0.186	0.878	0.061	0.348
	AK(mg/kg)	0.918	0.020	-0.234	0.193	0.203	0.977	0.068	0.428
	EC	0.714	0.346	0.426	0.242	0.331	0.979	0.068	0.465
	TS (g/kg)	-0.284	-0.569	0.551	-0.107	0.413	0.890	0.062	0.434
	SWC (%)	-0.298	0.823	0.276	-0.256	-0.007	0.909	0.063	0.333
	pH	0.600	0.722	-0.229	0.119	0.124	0.963	0.067	0.611
	eigenvalue	5.177	4.488	2.181	1.320	1.182			
	Percentage of Variance (%)	34.511	29.917	14.541	8.801	7.882			
	Cumulative (%)	34.511	64.429	78.970	87.771	95.652			

		principal component			Common factor variance	Weight	Membership degree
		PC1	PC2	PC3			
F. krylovii Korovin	SOM (g/kg)	-0.157	0.979	-0.062	0.988	0.072	0.498
	TN (g/kg)	0.665	0.441	0.597	0.994	0.072	0.325
	TP (g/kg)	0.823	0.102	-0.467	0.905	0.066	0.330
	C:N	-0.776	0.048	-0.617	0.984	0.072	0.654
	C:P	-0.687	0.641	0.231	0.936	0.068	0.600
	N:P	0.326	0.427	0.837	0.990	0.072	0.396
	TK (g/kg)	0.938	0.029	0.131	0.898	0.065	0.419
	NO_3-N (mg/kg)	0.904	-0.273	-0.055	0.896	0.065	0.442
	NH_4-N(mg/kg)	0.709	-0.591	0.206	0.894	0.065	0.476
	AP(mg/kg)	0.816	0.293	-0.471	0.973	0.071	0.512
	AK(mg/kg)	0.789	0.574	-0.181	0.983	0.072	0.506
	EC	0.869	-0.076	-0.304	0.853	0.062	0.472
	TS (g/kg)	-0.579	0.361	-0.004	0.465	0.034	0.312
	SWC (%)	-0.015	-0.973	0.205	0.989	0.072	0.461
	pH	-0.351	-0.917	0.119	0.977	0.071	0.357
	eigenvalue	7.025	4.522	2.179			
	Percentage of Variance (%)	46.831	30.148	14.529			
	Cumulative (%)	46.831	76.979	91.507			

		principal component				Common factor variance	Weight	Membership degree
		PC1	PC2	PC3	PC4			
F. sinkiangensis	SOM (g/kg)	-0.725	0.028	0.180	0.547	0.859	0.063	0.507
	TN (g/kg)	0.643	-0.514	0.523	0.193	0.989	0.072	0.375
	TP (g/kg)	0.619	-0.496	-0.355	0.460	0.966	0.070	0.448
	C:N	-0.813	0.406	-0.374	0.017	0.966	0.070	0.623
	C:P	-0.857	0.274	0.330	0.180	0.950	0.069	0.463
	N:P	0.523	-0.418	0.717	0.068	0.966	0.070	0.376
	TK (g/kg)	0.783	0.178	-0.352	-0.290	0.853	0.062	0.600
	NO_3-N (mg/kg)	-0.655	-0.397	-0.422	0.258	0.831	0.061	0.629
	NH_4-N(mg/kg)	-0.173	0.769	0.300	0.240	0.769	0.056	0.296
	AP(mg/kg)	0.403	-0.237	-0.310	0.822	0.990	0.072	0.298
	AK(mg/kg)	0.825	0.297	0.292	0.197	0.893	0.065	0.424
	EC	-0.826	-0.303	0.033	0.276	0.851	0.062	0.507
	TS (g/kg)	-0.814	0.030	0.476	0.037	0.891	0.065	0.409
	SWC (%)	0.441	0.733	-0.066	0.481	0.967	0.070	0.418
	pH	0.664	0.716	-0.004	0.167	0.982	0.072	0.480
	eigenvalue	6.886	2.993	1.999	1.844			
	Percentage of Variance (%)	45.905	19.955	13.328	12.293			
	Cumulative (%)	45.905	65.860	79.188	91.481			
